# Supplementary material for: Arrayed CRISPR libraries for the genome-wide activation, deletion and silencing of human protein-coding genes
Source: Nat Biomed Eng. 2024 Dec 4;9(1):127–48. doi: 10.1038/s41551-024-01278-4 (PMC11754104; doi:10.1038/s41551-024-01278-4)
Supplement: Supplementary file 1 — Supplementary Methods and Figures. [file 41551_2024_1278_MOESM1_ESM.pdf]

nature biomedical engineering

Supplementary information https://doi.org/10.1038/s41551-024-01278-4

Arrayed CRISPR libraries for the genome-wide activation, deletion and silencing of human protein-coding genes

In the format provided by the authors and unedited

Supplementary Methods

Primer information for real-time quantitative PCR

Gene	Forward primer sequence 5'-3'	Reverse primer sequence 5'-3'
ACTB	CATGTACGTTGCTATCCAGGC	CTCCTTAATGTCACGCACGAT
CXCR4	ACTACACCGAGGAAATGGGCT	CCCACAATGCCAGTTAAGAAGA
NEUROD1	GGATGACGATCAAAAGCCCAA	GCGTCTTAGAATAGCAAGGCA
LINC00925	AATTGTCCTGTGAAGTGAAG	TTCCTCTGTCTCCATTGTCA
TINCR	GGTCTGGGCTCCCAGGTGGA	TGTCAGGGACTGGGGCTCC
POU5F1	TATTTGGGAAGGTATTCAGC	CTTACACATGTTCTTGAAGC
KLF4	CTGGCGGGAGGAGCTCTCC	CGGCTCCGCCGCTCTCCA
LIN28A	GGGATGGATATATGAAGTAAGG	TAGCTACCATGACACTATTAAT
IL1R2	GCTGCCAGAAGCTGCCG	CTCAGGGCTACAGGCTCCC
IL1B	TCCTTCCAGGACCTGGACCT	CGGCCTGCCTGAAGCCC
MYC	CTGAGGAGGAACAAGAAGATGAGG	GTGGCCTCCAGCAGAAGG
NANOG	ccgtctctGGCTATAGATAAGTAG	ATCTTCATCACCAATTTGTACTTG
EGFR	GTTTGCCAAGGCACGAGTAA	GAGAAAATGATCTTCAAAAGTGCCC
HBG1	AATGTGGAAGATGCTGGAGG	GCCAAAGCTGTCAAAGAACC
TERT	CGGGCTGCTCCTGCGTTT	ATAGCTGAGGAAGGTTTTCGCG
ZFP42	CAGGTGTTTGCTGAAGACAG	GTTGCTGGCTCATGTTTTCC
LINC00028	CACTCCCACACCCCAAC	TGCCAACTTTCCACAGCTAG
LINC00514	AGAAGTGGTTTGGGCGC	CCGTTTCCATTGTTGTATCCTG
ASCL1	CGCGGCCAACAAGAAGATG	CGACGAGTAGGATGAGACCG
GAPDH	ATGATCTTGAGGCTGTTG	CTCAGACACCATGGGGAA
AGER	TGGATGAAGGATGGTGTGCC	CACAGCTGTAGGTTCCCTGG
APOE	CTGCTCAGCTCCCAGGTC	TTGTTCCTCCAGTTCCGATT
F2R	CCGCAGGCCAGAATCAAAAG	ACAAAGAGTGTCAGCCAGGAG
HES7	CCCCAAGATGCTCAAGCCG	GGTTCCGGAGGTTCTGGTC
LPAR4	GGCGGTATTTCAGCCTCTTT	AGCAGGTGGTGGTTGCATTG
ARG1	TCCCGATGTGCCAGGATTCT	ACGTCTCTCAAGCCAATATA
APP	CTCGTCACGTGTTCAATATG	GGGTGTGCTGTCTGTCCTTC
HMBS	GAAGGATGGGCAACTGTACC	ATGGTAGCCTGCATGGTCTC
PRNP	GTGCACGACTGCGTCAAT	CCTTCCTCATCCCACTATCAGG
SOX2	CAGCAAATGACAGCTGCAAA	ATCTCTCTCATAAAAGTTTTCTTGTC
VEGFA	AGATTATGCGGATCAAACCTCAC	CTGTAGGAAGCTCATCTCTCCT
LTF	ATGGTGGTTTCATATACGAGGCA	CTTTCGGTCCCGTAGACTTCC
ALX3	GGAGAAGACCTCCAAAGCTGC	gtCTCTGGGGCCCCCTCG
PBX1	CATGCTGTTAGCGGAAGGC	CTCCACTGAGTTGTCTGAACC
ZFY	AGAAAGCACATGCGAATCCAT	CGCACATCTCACACTTATGAGGA
PPARA	ATGGTGGACACGGAAAGCC	CGATGGATTGCGAAATCTCTTGG
MIXL1	GGCGTCAGAGTGGGAAATCC	GGCAGGCAGTTCACATCTACC
ZNF121	CTCATGCCTTAATGCACACATGG	AGGATTTGTCTCCTATCCACGTT
KLF12	CGGCAGTCAGAGTCAAAACAG	CGGCTTCCATATCGGGATAGT
ZNF678	TTACAGGGACTACTGGCATTCA	CAGCTTCTATGTCTTGTCAGTGT
CLOCK	TGCGAGGAACAATAGACCCAA	ATGGCCTATGTGTGCGTTGTA
NEUROD4	GAGAGCTAGTCAACACACCATC	GCATCCCATAAGTACCTGGTCTG
ZBTB33	TGCTGAACTCCTTGAATGAGC	CGGAATTTTCGGTCTTCCACAA
ZNF578	GTAAGGAAGAATTAACTGTCAGG	GAGAAACCTCTTTCACAAACCT
CHCHD3	GAGGCGGACGAGAATGAGAAC	ACCAGAATACCGCTGAGACTTC
SETDB2	TGCTCTGGTGCTTGTCTGATG	TGCATGTCGTCTTTGGAAGTG
GLIS3	GTTCAGCGACTGGGACTCATT	CCCTCTGTAAGCTAGGACTGAT
ZNF821	AAGAGGAGACGACACAAGATGA	GCTCTACCACTTCGTTCCCAC
ZBTB38	ACACTTGCCGAGCACTCATAC	CCCTGTGTTACCAGGTGAACT

SIX5	TTGGCGCAGTGGACAAGTATC	AAGCAGTAGACTGTCTCCTCG
ZFP1	ACCCACCAGTCAAACCTCATT	AGGGTCGCTCTCCTGTATGAA
ZNF260	ATGTGGTAAAGTGTGCTCTCG	GGTGTCTGATGATGGTTGGCA
ZNF583	CCATGCcctgattgggagtatg	CTGGGAtagtcaaggctataac
BNC2	AAATCAGAGGACAGGCTTAGTGA	TTGAGATGTATCAACCCCACAAC
ZBTB9	CCACGGACAATCCAGATCGAG	CTTTATGAGCCCTAAGTTCCCG
ZNF445	TGCTGCCTATCCAGCTCAG	GGCGGTTGAGAGTCTGTGG
ZNF112	GGAACCTGCTCTTAGTAGCACA	CGGGAGGAAAGCTCTTTGGG
NR2F6	GAGCGGCAAGCATTACGGT	GGCAGGTGTAGCTGAGGTT
HNF4A	CGAAGGTCAAGCTATGAGGACA	ATCTGCGATGCTGGCAATCT
GLI3	GAAGTGCTCCACTCGAACAGA	GTGGCTGCATAGTGATTGCG
ZNF227	GCTTCCCCTTCATCCGAATGT	GCAACTGTCGCATCTATAAGACT
ZNF331	TTCGCCGACGTAGCCATAGA	CGTCCCAGTACAGGTCCCT
ZNF81	GCAGTGCCTGTGAGGTATCAG	CGTCTTTGAGTAGAGTCCAGTTG
TFEB	ACCTGTCCGAGACCTATGGG	CGTCCAGACGCATAATGTTGTC
ESRRB	ATCAAGTGCGAGTACATGCTC	CGCCTCCGTTTGGTGATCTC
ZNF519	GGAAATGCCTAGACCCTGCC	GCTCTGGTAAAATGCCTTGGTTG
E2F3	AGAAAGCGGTCATCAGTACCT	TGGACTTCGTAGTGCAGCTCT

Primer information for SMRT sequencing of genome-edited HEK293 cells

Primer name	Primer sequence (5′-3′, lowercase nucleotides indicate barcodes, uppercase nucleotides indicate annealing sequence to the genome)
APEX1 Fwd bc1	aaacaaacacGTCTGTAGGCAACGCGGTA
APEX1 Fwd bc2	aaacaagggtGTCTGTAGGCAACGCGGTA
APEX1 Fwd bc3	aaacatctctGTCTGTAGGCAACGCGGTA
APEX1 Fwd bc4	aaaccctgacGTCTGTAGGCAACGCGGTA
APEX1 Rev bc1	aaacgggaacTTTGTTCCCCTTGGGGTTGC
APEX1 Rev bc2	aaattgtacgTTTGTTCCCCTTGGGGTTGC
TAZ Fwd bc1	aacagatgaaGAGGGGGATAGTCCCCAACA
TAZ Fwd bc2	aaccttagatGAGGGGGATAGTCCCCAACA
TAZ Fwd bc3	aactacttgcGAGGGGGATAGTCCCCAACA
TAZ Fwd bc4	aagaacgccgGAGGGGGATAGTCCCCAACA
TAZ Rev bc1	aagcgtgtgaACTGAAGGGCTTCCCGATCA
TAZ Rev bc2	acaaatcgagACTGAAGGGCTTCCCGATCA
PRNP Fwd bc1	acatgcaaccGCAGCTGATACCATTGCTATGC
PRNP Fwd bc2	acattcctgtGCAGCTGATACCATTGCTATGC
PRNP Fwd bc3	accacctaatGCAGCTGATACCATTGCTATGC
PRNP Fwd bc4	acccgttcgtGCAGCTGATACCATTGCTATGC
PRNP Rev bc1	accgagccatCACCACCACTAAAAGGGCTG
PRNP Rev bc2	actcaacaggCACCACCACTAAAAGGGCTG
CSNK2A1 Fwd bc1	actgcggaagGGGTTCACCAGGGTGACATTT
CSNK2A1 Fwd bc2	agaaggtcctGGGTTCACCAGGGTGACATTT
CSNK2A1 Fwd bc3	agaccttgttGGGTTCACCAGGGTGACATTT
CSNK2A1 Fwd bc4	agagctagcgGGGTTCACCAGGGTGACATTT
CSNK2A1 Rev bc1	agattactcaTGTAACTCGGCAAAGACGGT

CSNK2A1 Rev bc2	agcataagtaTGTAACTCGGCAAAGACGGT
TGFBR1 Fwd bc1	agccgtcacaTCCTGCCTAACCACCGTACT
TGFBR1 Fwd bc2	agggccgattTCCTGCCTAACCACCGTACT
TGFBR1 Fwd bc3	aggtcttccgTCCTGCCTAACCACCGTACT
TGFBR1 Fwd bc4	agtaacacccTCCTGCCTAACCACCGTACT
TGFBR1 Rev bc1	agtcagtggcAGCCCTTGGCAACTCAGAAC
TGFBR1 Rev bc2	agtggtttaaAGCCCTTGGCAACTCAGAAC
HPRT1 Fwd bc1	attagctgggCACGTGTGAACCAACCCGCC
HPRT1 Fwd bc2	attctgcatcCACGTGTGAACCAACCCGCC
HPRT1 Fwd bc3	caatacgaatCACGTGTGAACCAACCCGCC
HPRT1 Fwd bc4	cacgagttaaCACGTGTGAACCAACCCGCC
HPRT1 Rev bc1	cactctcaggCTGGTCCCTACAGAGTCCCA
HPRT1 Rev bc2	cagggatggtCTGGTCCCTACAGAGTCCCA
AP2B1 Fwd bc1	cagtgaccagTGAGGCTTCTGCATTGTTGGA
AP2B1 Fwd bc2	catcacctttTGAGGCTTCTGCATTGTTGGA
AP2B1 Fwd bc3	catcatagacTGAGGCTTCTGCATTGTTGGA
AP2B1 Fwd bc4	cattggtgccTGAGGCTTCTGCATTGTTGGA
AP2B1 Rev bc1	ccaaagttgcAACTGTTAGCCTCCTGGTGC
AP2B1 Rev bc2	ccaacccacaAACTGTTAGCCTCCTGGTGC
ADIPOR1 Fwd bc1	ccaggctcttGGACTCTGCTAGCCCCATTC
ADIPOR1 Fwd bc2	cccatttagcGGACTCTGCTAGCCCCATTC
ADIPOR1 Fwd bc3	cccgaaagagGGACTCTGCTAGCCCCATTC
ADIPOR1 Fwd bc4	ccgtattcgaGGACTCTGCTAGCCCCATTC
ADIPOR1 Rev bc1	cctccgtactAGTGCACTGGCACTCTAAGC
ADIPOR1 Rev bc2	cctggcaaatAGTGCACTGGCACTCTAAGC
FYN Fwd bc1	cctttgaagtGGGGAGGAAAACAACCCACA
FYN Fwd bc2	cgaaattcgtGGGGAGGAAAACAACCCACA
FYN Fwd bc3	cgactaaaccGGGGAGGAAAACAACCCACA
FYN Fwd bc4	cgagggcaatGGGGAGGAAAACAACCCACA
FYN Rev bc1	cgctttatacAGCTGTGGGTTGTGTAAGTCT
FYN Rev bc2	cggagagcttAGCTGTGGGTTGTGTAAGTCT

High-throughput lentiviral production in 384-well plates

Cell seeding. A monoclonal HEK293T cell line that produces higher titters of lentivirus was generated in-house and used for high-throughput lentiviral production. 6,800 cells were seeded with Integra Viaflo in 50 µl of DMEM + 10% FBS medium per well.
Plasmid transfection. Around 24 hours after seeding, when cells reached 80–90% confluency, the mix was prepared according to the following table (the amount was for three 384-well plates of plasmid set, each plasmid set for three technical repeats, each plasmid set a 384-well plate of plasmids) for transfection:

	Opti-MEM	psPAX2	VSV-G	P3000	Lipofectamine
Tube 1	14.7 ml	69.34 µg	46.23 µg	441.3 µl	
Tube 2	18.3 ml				441.3 µl

Cells were transfected with the following procedure within 1 hour after the mix had been prepared:

1. Tube 1 was prepared and 38 µl of the mix was transferred to each well of a 384-deep-well-plate using a multichannel pipette (plate with Mix 1).
2. 10.1 µl (per well) of the above-mentioned Mix 1 was then transferred to three 384-well PCR-plates using the Viaflo (12.5 µl-tips).
3. Tube 2 was prepared and 46 µl (per well) of the mix was transferred to a 384-deep-well-plate using a multichannel pipette.
4. 72 ng (2.4 µl of 30 ng/µl dilution) of qgRNA plasmid (from the library in 384-well-format) was added to the plate prepared at Step 2 and mixed for 10 cycles (Viaflo; speed up/down: 8), then incubated for 5 minutes at room temperature (RT).
5. 12.5 µl (per well) of the Lipofectamine 3000 mix prepared at Step 3 was transferred to the plates prepared at Step 4 to obtain 25 µl of transfection mix per well and mixed for 20 cycles (speed up/down: 8), then incubated for 20 minutes at RT.
6. 36 µl of medium was removed from the cell culture plate (ViaFlo setting: 31.5 mm; speed up 3).
7. The plate prepared at Step 5 was centrifuged at 1500 rpm for 2 minutes.
8. 8 µl of transfection mix was added to each replicate of the three replicate cell culture plates (ViaFlo setting: 16 mm; speed down 3); and incubated at 37 °C for 6–12 hours.

Virus harvesting medium addition. Virus Harvesting Medium (composition for a 500 ml: 434 ml of DMEM + 59 ml of 10% FBS + 6.82 grams of BSA + 6.8 ml of penicillin or streptomycin) was prepared and warmed up to 37 °C. Then 44 µl per well of Virus Harvesting Medium was added to the plates with transfected cells (ViaFlo settings: 37mm, speed up: 8, speed down: 2).

Virus collection. 48–60 hours after the addition of the virus harvesting medium, check the transfection efficiency of the cells by visual inspection the percentage of TagBFP positive cells, a less than 50% transfection efficiency indicates the failure of viral production. If transfection efficiency is 50% or higher, 45 µl of viruses of each replicate transfected plate was collected, and viruses from the three replicates of the same plasmid set were pooled together, mixed for 20 cycles and then centrifuged at 1500 rpm for 2 minutes to pellet cells. Viral supernatant was aliquoted into PCR plates for storage at −80°C, for later use and titter determination.

Virus titration. One day before virus titration, 6,000 HEK293T cells were seeded in 384-well plates. Lentiviruses produced in 384-well plates were thawed from −80 °C and 0.5 µl of viruses (per well) were transduced into the HEK293T cells. Three days post-transduction, cell culture medium was removed, and cells were dissociated with 15 µl of PBS-EDTA (2 mM EDTA in 1× PBS) for 15–20 min at 37 °C. Later, 20 µl of FACS buffer (10 mM EDTA + 5% FBS in 1× PBS) was added to each well and mixed with the Viaflo until a single-cell suspension was obtained. Then the percentage of TagBFP-positive cells was analysed by flow cytometry. If 90% of wells in the plate reaches a virus titter of 10^6 transuding unit per ml, the plate of virus is considered a successful lentiviral production. Otherwise, the plate needs to be reproduced until filling the criterion. Our protocol reaches 81.5% successful rate for the T.gonfio library lentiviral packaging. The success rate is calculated by dividing the number of successful plates by the total number of plates produced during the procedure of virus production.

ALPA cloning high-throughput generation of libraries

Oligo synthesis**.** Twenty-nucleotide sgRNA sequences were incorporated into oligonucleotide sequences with appended constant sequences and synthesized in 384-well plates using the high affinity purification (HAP) purification method by Sangon Biotech (China). The sgRNA1 (sgRNA1 sequence, N_{20sg1}) oligonucleotide sequence is: 5′-ttgtggaaaggacgaaacaccGN_{20sg1}GTTTAAGAGCTAAGCTG-3′; the sgRNA2 (sgRNA2 sequence, N_{20sg2}) oligo sequence is: 5′- cttggagaaaagccttgtttGN_{20sg2}GTTTGAGAGCTAAGCAGA-3′; the sgRNA3 (sgRNA3 sequence, N_{20sg3}) oligo sequence is: 5′ -gtatgagaccactctttcccGN_{20sg3}GTTTCAGAGCTAAGCACA-3′; and the sgRNA4 (reverse complement sequence of sgRNA4, $N_{20\ crsg4}$) oligo sequence is 5′ -ATTTCTGCTGTAGCTCTGAAAC$N_{20crsg4}$Cgaggtacccaagcggc-3′. The oligonucleotides were diluted with ddH_2O to a working concentration of 4 µM.

Three-fragment PCRs. A total of 10 µL PCR reaction per well was produced in 384-well plates. The C1 fragment (amplicon size 761 bp) PCR mix was prepared as follows:

Volume/well	Reagent
0.2 µL	C1 fragment 1 ng/µL
0.2 µL	mU6 Rev primer 10 µM<br>(common primer, sequence attached)
2 µL	5X HF buffer
0.2 µL	dNTPs 10 mM
0.1 µL	Phusion High-Fidelity DNA polymerase
6.8 µL	ddH_2O

9.5 µL of the mix were aliquoted in each well of the 384-well plate, and 0.5 µl of sgRNA1 primer (at 4 µM concentration) was added to each well and mixed.

The M fragment (amplicon size 360 bp) PCR mix was prepared as follows:

volume/well	Reagent
0.2 µL	M fragment 1 ng/µL
0.2 µL	M Rev primer 10 µM<br>(common primer, sequence attached)
2 µL	5X HF buffer
0.2 µL	dNTPs 10 mM
0.1 µL	Phusion High-Fidelity DNA polymerase
6.8 µL	ddH_2O

9.5 µL of the mix were aliquoted in each well of the 384-well plate, and then 0.5 µl of sgRNA2 primer (at 4 µM concentration) was added to each well and mixed.

C2s fragment (amplicon size 422 bp) PCR mix was prepared as follows:

vol/well	Reagent
0.2 µL	C2s fragment 1ng/µL
2 µL	5X HF buffer
0.2 µL	dNTPs 10 mM
0.1 µL	Phusion High-Fidelity DNA polymerase
6.5 µL	ddH_2O

9 µL of the mix were aliquoted in each well of the 384-well plate, and then 0.5 µL of sgRNA3 primer (at 4 µM concentration) and 0.5 µL of sgRNA4 primer (also at 4 µM concentration) were added to each well and mixed.

The Integra ViaFlo 384-well pipetting system was used for all 384-well liquid handling. All PCR plates were sealed tightly and centrifuged at 2000 rpm for 2 minutes, and placed in thermocyclers with the following program: Preheat the lid at 99 °C; Initial denaturation at 98 °C for 30 seconds, 36 cycles comprising 98 °C for 10 seconds, 60 °C for 30 seconds, and 72 °C for 25 seconds, and final extension at 72 °C for 5 minutes, followed by cooldown to 20 °C. All PCR products were then diluted with 9 µL of ddH_2O for later Gibson assembly. The success of PCR on each plate was confirmed by DNA agarose gel electrophoresis of several random samples on the plate.

Gibson assembly. Assembly of the three fragment PCR products into the pYJA5 vector was performed in a 384-well plate by Gibson assembly, with the following reaction mix:

Volume	Reagent

2 µL	C1 amplified fragment (estimated around 16 ng/µL)
1 µL	M amplified fragment (estimated around 16 ng/µL)
1 µL	C2s amplified fragment (estimated around 20 ng/µL)
1 µL	pYJA5 *Bbs*I digested purified vector, diluted to 120 ng/µL
5 µL	2X homemade HiFi Gibson master mix

The mix was incubated in the thermocycler at 50 °C for 1 hour, and then used for the transformation of competent cells or stored immediately at −20 °C.

In silico qgRNA libraries design

Pooling existing libraries. To provide a starting point for guide RNA selection, we collected sgRNAs from previously published and validated libraries and tools, each of which employed their own algorithms to select sgRNAs with high predicted on-target efficacy. We included the Calabrese[1] and hCRISPRa v2(ref.[2]) libraries for T.gonfio, and the TKOv3(ref.[3]) and Brunello[1, 4] libraries for T.spiezzo. We complemented these source libraries with sgRNAs from the CRISPick tool (formerly GPP sgRNA Designer, https://portals.broadinstitute.org/gppx/crispick/public), to ensure optimal coverage of difficult-to-target and newly annotated genes (the website was accessed in April 2020, following the update of 20 March 2020).

Gene definitions. Entrez gene identifiers were used to provide common gene definitions for sgRNAs from all sources. If the source library did not provide Entrez identifiers, the official gene symbols were mapped to Entrez IDs, and the genomic location was used to disambiguate gene symbols when necessary. Genes that were not defined as protein-coding by NCBI or Ensembl were excluded according to the following annotation files:

ftp://ftp.ncbi.nih.gov/gene/DATA/GENE_INFO/Mammalia/Homo_sapiens.gene_info.gz
ftp://ftp.ensembl.org/pub/release-99/tsv/homo_sapiens/Homo_sapiens.GRCh38.99.entrez.tsv.gz

Both files were downloaded on 25 March 2020. The final libraries included 19839 protein-coding genes for T.gonfio and 19820 for T.spiezzo; the difference in gene counts arises from genes that were present for only one modality in our source libraries. For example, highly polymorphic genes related to adaptive immunity, such as the T Cell Receptor Alpha Locus (TRA) gene, are available for T.gonfio, but not T.spiezzo.

Transcriptional start site (TSS) definitions. To ensure good coverage of alternative transcripts and the broad applicability of the T.gonfio library in multiple cell lines, we adopted the alternative transcription start site (TSS) definitions from the hCRISPRa-v2 library[2]. The authors of this library used the FANTOM5 CAGE-seq dataset[5], supplemented by Ensembl[6] transcript models, to define TSS positions. Additional TSSs were targeted by a separate set of sgRNAs if the FANTOM5 scores indicated significant transcriptional activity, and if they were spaced more than one kilobase apart from the primary TSS. We chose a separate set of four sgRNAs for each TSS, treating multiple TSSs as if they were distinct genes. To group sgRNAs by TSS, we mapped sgRNAs from all sources (including the top five sgRNAs from the CRISPick sgRNA Designer) to their genomic locations. We then iterated through each sgRNA, starting with the lowest genomic coordinate; a new TSS group was defined if the distance from one guide to the next exceeded 1000 base pairs. Additional TSSs were only targeted if a valid combination of four guides was available. Multiple TSSs were included for 2,311 genes using 4,803 four-guide combinations, whereas a single TSS was targeted for the remaining 17,528 genes.

Avoidance of genetic polymorphisms. For each sgRNA, we checked for overlaps with regions of frequent genetic polymorphism in human populations. We considered both the 20-nucleotide target sequence and the two guanosine nucleotides of the NGG PAM; however, the first nucleotide of the PAM was allowed to vary. We avoided sgRNAs whose target region contained any genetic polymorphisms with frequencies greater than 0.1%. Variant frequencies were derived from the Kaviar

database[7], which includes curated genomic data on single nucleotide variants, indels, and complex variants from over 77,000 individuals (including over 13,000 whole genomes). The dataset (only variants seen more than 3 times, version 160204-hg38) was downloaded on 7 August 2019. The polymorphism frequencies in the Kaviar database were generally similar to those from TOPMED, gnomAD, and the 1000 Genomes Project.

Specificity scores. To select a four-guide combination with minimal off-target effects, we computed specificity scores for each sgRNA from our source libraries. We used the approach introduced by the authors of the GuideScan[8] tool: For each guide, potential off-target sites were weighted by their CFD (cutting frequency determination) scores[4], and CFD scores were aggregated into a single score using the formula: 1 / (1 + sum of CFD scores from all off-target sites[9]. Because the pre-computed GuideScan Cas9 database does not contain all sgRNAs (it excludes those with perfect-match or one-mismatch off-target sites in the reference genome), we annotated sgRNAs using both the GuideScan and CRISPOR[10, 11] tools. Local installations of these tools were used, and the source code was downloaded in December 2020 (GuideScan version 2018-05-16, and CRISPOR version 4.97). The output of the local installations was confirmed to be identical to that of the web-based tools. When available, we used GuideScan specificity scores (considering up to three mismatches); otherwise, CRISPOR specificity scores were used (considering up to four mismatches). CRISPOR three-mismatch (3MM) and four-mismatch (4MM) specificity scores analogous to those from GuideScan were computed, using the detailed output files listing each off-target site. GuideScan and CRISPOR specificity scores were highly correlated, but not identical, due to slight differences in the number of off-target sites identified for the same sequence. When selecting sgRNAs, we avoided low-specificity guides with 3MM scores below 0.2; this cut-off point was recently shown to have good predictive power for identifying sgRNAs with significant off-target activity[12]. However, this criterion had to be relaxed in cases where all eligible sgRNAs had specificity scores below 0.2, for example, when targeting genes present in multiple copies in the genome, or those belonging to large gene families with many closely related paralogs and pseudogenes. Finally, to choose among all eligible four-guide combinations, we computed an aggregate specificity score using the formula: 1 / (1 + sum of CFD scores from all four guides), and picked the combination with the highest score, indicating high predicted specificity.

sgRNA spacing. To allow for unhindered multiple binding for synergistic effect, we selected, whenever possible, four sgRNAs whose “cut” locations were spaced at least 50 base pairs apart. However, for CRISPRa, target sequences should be located within a window of about 400 base pairs upstream of the TSS for optimal activity[13], which is reflected in the selection of sgRNAs in the source libraries. Thus, overlaps were unavoidable for some genes. For CRISPRko, on the other hand, overlaps were often inevitable when targeting genes with very short coding sequences. In those cases, we nevertheless aimed to minimize the total number of overlaps between neighbouring guides. Furthermore, all four-guide combinations strictly adhered to another criterion: No two sgRNAs were allowed to share identical sub-sequences of more than seven base pairs. This was done primarily to minimize recombination events between identical regions during Gibson assembly of the plasmid. However, this also enforced minimal spacing of the four selected guides.

Selection of four sgRNAs. After integration and annotation of sgRNAs from the source libraries, we selected the final combination of four sgRNAs for each gene or TSS. First, sgRNAs containing a stretch of four or more T nucleotides were excluded, since this sequence can induce termination of transcription. Next, all possible four-guide combinations for each gene were generated, and combinations that shared identical subsequences greater than seven base pairs in length were excluded. The potential combinations were then ranked, using a list of criteria that were applied in order; if multiple combinations were tied in first place, the decision was made using the next criterion down the list. The criteria were as follows: 1) Maximize the number of sgRNAs (from zero to four) that fulfil certain minimal requirements – the sgRNA can be mapped to a defined genomic location in the reference genome with an N(GG) PAM; there are no overlaps with frequent genetic polymorphisms (>0.1%); the 3MM specificity score is at least 0.2; and for T.spiezzo only, the guide conforms to the criteria of ref.[14]; 2) maximize the number of sgRNAs with exactly one perfect match location in the reference genome, 3) minimize the number of overlaps between two neighbouring sgRNAs spaced fewer than 50 base pairs apart, 4) minimize the number of sgRNAs derived from the CRISPick sgRNA

Designer tool, rather than the previously published libraries, 5) for T.gonfio, minimize the number of sgRNAs derived from the “supplemental 5” rather than “top 5” sgRNAs for the hCRISPRa-v2 library, and for T.spiezzo, minimize the number of CRISPick-derived sgRNAs ranked outside the top 10, and 6) maximize the aggregate specificity score from all 4 guides. The highest-ranked four-guide combination was chosen. Since the aggregate specificity score was the only quantitative criterion, it acted as a tiebreaker and had the greatest impact on the choice of guides.

Sublibrary allocation. To facilitate focussed screens of a subset of the genome, we divided the entire set of protein-coding genes into mutually exclusive sub-libraries. Two of our sub-libraries – Transcription Factors, and Secretome – were based on recent publications that combined bioinformatics analyses with expert curation to arrive at a comprehensive list of genes in those categories[15, 16]. These lists were obtained from the publication’s supplemental data (for the secretome) or the authors’ website (for the transcription factors; humantfs.ccbr.utoronto.ca, database version 1.01). Ensembl gene IDs were translated to Entrez gene IDs, making use of HUGO gene symbols to disambiguate one-to-many mappings for a few genes. A third sub-library was based on a list of G-protein coupled receptors, curated by the HUGO Gene Nomenclature Committee (HGNC)[17] (https://www.genenames.org/cgi-bin/genegroup/download?id=139&type=branch, accessed on 11 Marc 2020). An additional seven thematic sub-libraries were adopted from the hCRISPRa-v2 library[2]: Membrane Proteins, Kinases/Phosphatases/Drug Targets, Mitochondria/Trafficking/Motility, Stress/Proteostasis, Cancer/Apoptosis, Gene Expression, and Unassigned. The first two of these thematic sub-libraries were updated to incorporate a small number of additional transmembrane receptors, transporters, kinases and phosphates, using Gene Ontology terms (exported from BioMart[18] on 25 March 2020) and a list of membrane proteins provided by the Human Protein Atlas project[19] (https://www.proteinatlas.org/search/protein_class:Predicted+membrane+proteins, accessed on 11 March 2020). If a gene belonged to multiple categories, it was assigned to the first sub-library (in the order in which they are listed in this section), and all remaining genes were added to the Unassigned sub-library.

Classification of unintended gene perturbations. Some sgRNAs are expected to perturb additional genes other than the intended target gene. In certain cases, this occurs at a different locus than the intended target site: For example, gene families of very close paralogs can often only be targeted with sgRNAs that have multiple perfect-match binding sites in the genome. However, in most cases, this involves a single locus – the intended binding site – where a sgRNA may perturb more than one gene. In the case of T.gonfio, the same promoter region is often shared by two genes located on opposite strands of the chromosome, so that their transcription start sites (TSSs) lie only a few hundred base pairs apart. In this case, guide RNAs that effectively activate one gene would inevitably also activate the other. As a guide for users of the library, and to aid the interpretation of hit genes, we annotated sgRNAs with a complete list of all genes they target. For the purpose of summarizing this phenomenon across the entire library, we classified each sgRNA as 1) only targeting the intended gene, 2) targeting unintended genes, but in a single location (on-site off-target effects), or 3) targeting unintended genes at other locations (off-site off-target effects). If two perfect-match sgRNA binding sites had any target genes in common, they were considered to target unintended genes at the same location. This ensured that sgRNAs targeting the pseudoautosomal region of chromosomes X and Y were classified correctly. Genes in this region technically map to two different chromosomes, but they are present in only two copies in diploid cells, just like any other gene. Unless they have additional target sites, they should not be included in the “off-site” category.

Annotation of unintended target genes. To annotate each sgRNA with all its potential target genes, a database of TSS locations was constructed by merging the FANTOM5 dataset (lifted over to the hg38 genome[20], version 3) with data from BioMart[18] (exported on 25 March 2020), using Entrez gene IDs as a common identifier. Similarly, data on coding sequence (CDS) and exon locations were compiled from BioMart, the “TxDb.Hsapiens.UCSC.hg38.knownGene” Bioconductor package (version 3.10.0), and GENCODE[21] annotation data (Release 33), and location data were merged using Entrez gene identifiers (if available) or Ensembl gene identifiers. Genes annotated as pseudogenes, or whose categorization was unclear, were excluded from further analysis. For T.gonfio, perfect-match sgRNA binding sites within a window of 1000 base pairs around TSSs were considered. For

T.spiezzo, sgRNA cut locations had to lie within the coding sequences (CDSs) of protein-coding genes, or within the exons of non-coding RNAs.

Annotation of predicted deletions. In the case of T.spiezzo, when four sgRNAs are active within the same cell, the multiple, closely spaced double-strand breaks commonly lead to the loss of a DNA segment between the sgRNA cut locations. Thus, in addition to annotating individual sgRNAs, we also determined which genes are affected by the predicted deletion – the segment between the first and last cut site. We also took deletions induced by (perfect-match) off-target binding sites into consideration. Because deletions may be less likely to occur if the cut sites are very far apart, we imposed a maximum distance of one megabase between cut sites, so that multiple predicted deletions (or isolated cut positions) on the same chromosome were possible.

Primer information for SMRT long-read sequencing of the libraries
The barcoded primers were HPLC-purified and are listed below (lowercase nucleotides indicate barcodes, uppercase nucleotides indicate the sequence annealing to the plasmid vector):

Row	Row primers Fwd sequence (5′-3′)	Column	Column primers Rev sequence (5′-3′)
2PF_A	acgctaacggAGTACCGGGCCCTACGCGTT	2PR_1	tgagctcacgCGGAGCCGGTTGGCGCCTAC
2PF_B	gcgattctatAGTACCGGGCCCTACGCGTT	2PR_2	gaacttattgCGGAGCCGGTTGGCGCCTAC
2PF_C	aagattgcacAGTACCGGGCCCTACGCGTT	2PR_3	tatgagtagtCGGAGCCGGTTGGCGCCTAC
2PF_D	attgcgtcaaAGTACCGGGCCCTACGCGTT	2PR_4	gatttgtaagCGGAGCCGGTTGGCGCCTAC
2PF_E	ctcataagatAGTACCGGGCCCTACGCGTT	2PR_5	agttgtcagtCGGAGCCGGTTGGCGCCTAC
2PF_F	gtctcaacggAGTACCGGGCCCTACGCGTT	2PR_6	tagctcgttcCGGAGCCGGTTGGCGCCTAC
2PF_G	catgcgcaacAGTACCGGGCCCTACGCGTT	2PR_7	aggtaatagtCGGAGCCGGTTGGCGCCTAC
2PF_H	ttgtgaagtgAGTACCGGGCCCTACGCGTT	2PR_8	tgcccacacaCGGAGCCGGTTGGCGCCTAC
2PF_I	tctactatccAGTACCGGGCCCTACGCGTT	2PR_9	acgtcaaaatCGGAGCCGGTTGGCGCCTAC
2PF_J	ttaaacgcatAGTACCGGGCCCTACGCGTT	2PR_10	aagagaacggCGGAGCCGGTTGGCGCCTAC
2PF_K	atctcgcacaAGTACCGGGCCCTACGCGTT	2PR_11	tcgtaacattCGGAGCCGGTTGGCGCCTAC
2PF_L	gtaactcccgAGTACCGGGCCCTACGCGTT	2PR_12	atccgtgattCGGAGCCGGTTGGCGCCTAC
2PF_M	aatatgtcggAGTACCGGGCCCTACGCGTT	2PR_13	ttctcactacCGGAGCCGGTTGGCGCCTAC
2PF_N	catctgatgtAGTACCGGGCCCTACGCGTT	2PR_14	tatcagtactCGGAGCCGGTTGGCGCCTAC
2PF_O	tgcccgggccAGTACCGGGCCCTACGCGTT	2PR_15	cacgactcgaCGGAGCCGGTTGGCGCCTAC
2PF_P	gagttagtgtAGTACCGGGCCCTACGCGTT	2PR_16	ctctgccgcaCGGAGCCGGTTGGCGCCTAC
		2PR_17	gccacgtatcCGGAGCCGGTTGGCGCCTAC
		2PR_18	cagtcagcgtCGGAGCCGGTTGGCGCCTAC
		2PR_19	taaggagtgcCGGAGCCGGTTGGCGCCTAC
		2PR_20	tctgagcaggCGGAGCCGGTTGGCGCCTAC
		2PR_21	acaactcgcgCGGAGCCGGTTGGCGCCTAC

		2PR_22	tagtgagagtCGGAGCCGGTTGGCGC CTAC
		2PR_23	tacatgttcgCGGAGCCGGTTGGCGCC TAC
		2PR_24	ccgacacggaCGGAGCCGGTTGGCG CCTAC

Illumina sequencing of T.spiezzo/T.gonfio pooled screen

A schematic of the sequencing strategy:

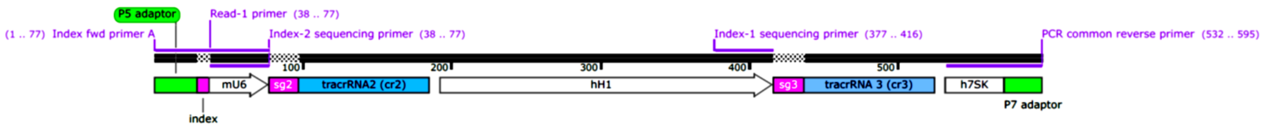

The primers used for gDNA amplification and sequencing:

Index-Fwd_A	AATGATACGGCGACCACCGAGATCTACACCAGTGCTTTTTGAGACTAT AAATATCCCTTGGAGAAAAGCCTTGTTTG
Index-Fwd_B	AATGATACGGCGACCACCGAGATCTACACTCCATTACTTTGAGACTATA AATATCCCTTGGAGAAAAGCCTTGTTTG
Index-Fwd_C	AATGATACGGCGACCACCGAGATCTACACGTCGACTGTTTGAGACTAT AAATATCCCTTGGAGAAAAGCCTTGTTTG
Index-Fwd_D	AATGATACGGCGACCACCGAGATCTACACAGTACGCCTTTGAGACTAT AAATATCCCTTGGAGAAAAGCCTTGTTTG
Index-Fwd_E	AATGATACGGCGACCACCGAGATCTACACTAGGCAGTTTTGAGACTAT AAATATCCCTTGGAGAAAAGCCTTGTTTG
Index-Fwd_F	AATGATACGGCGACCACCGAGATCTACACCTAATGGATTTGAGACTATA AATATCCCTTGGAGAAAAGCCTTGTTTG
Common-Rev	CAAGCAGAAGACGGCATACGAGATGAATGCCTTGCAGATGGGTGGGG CATGCTAAATACTGCAG
Read-1 primer (for forward sequencing sgRNA2)	TTTGAGACTATAAATATCCCTTGGAGAAAAGCCTTGTTTG
Index-1 sequencing primer (for forward sequencing sgRNA3)	TGGGAATCTTATAAGTTCTGTATGAGACCACTCTTTCCCG
Index-2 sequencing primer (for reverse sequencing index)	CAAACAAGGCTTTTCTCCAAGGGATATTTATAGTCTCAAA

References

1. Sanson, K.R. et al. Optimized libraries for CRISPR-Cas9 genetic screens with multiple modalities. *Nat Commun* **9**, 5416 (2018).
2. Horlbeck, M.A. et al. Compact and highly active next-generation libraries for CRISPR-mediated gene repression and activation. *Elife* **5** (2016).
3. Hart, T. et al. Evaluation and Design of Genome-Wide CRISPR/SpCas9 Knockout Screens. *G3 (Bethesda)* **7**, 2719-2727 (2017).
4. Doench, J.G. et al. Optimized sgRNA design to maximize activity and minimize off-target effects of CRISPR-Cas9. *Nat Biotechnol* **34**, 184-191 (2016).
5. Consortium, F. et al. A promoter-level mammalian expression atlas. *Nature* **507**, 462-470 (2014).
6. Yates, A.D. et al. Ensembl 2020. *Nucleic Acids Res* **48**, D682-D688 (2020).
7. Glusman, G., Caballero, J., Mauldin, D.E., Hood, L. & Roach, J.C. Kaviar: an accessible system for testing SNV novelty. *Bioinformatics* **27**, 3216-3217 (2011).
8. Perez, A.R. et al. GuideScan software for improved single and paired CRISPR guide RNA design. *Nat Biotechnol* **35**, 347-349 (2017).
9. Hsu, P.D. et al. DNA targeting specificity of RNA-guided Cas9 nucleases. *Nat Biotechnol* **31**, 827-832 (2013).
10. Haeussler, M. et al. Evaluation of off-target and on-target scoring algorithms and integration into the guide RNA selection tool CRISPOR. *Genome Biol* **17**, 148 (2016).
11. Concordet, J.P. & Haeussler, M. CRISPOR: intuitive guide selection for CRISPR/Cas9 genome editing experiments and screens. *Nucleic Acids Res* **46**, W242-W245 (2018).
12. Tycko, J. et al. Mitigation of off-target toxicity in CRISPR-Cas9 screens for essential non-coding elements. *Nat Commun* **10**, 4063 (2019).
13. Gilbert, L.A. et al. Genome-Scale CRISPR-Mediated Control of Gene Repression and Activation. *Cell* **159**, 647-661 (2014).
14. Graf, R., Li, X., Chu, V.T. & Rajewsky, K. sgRNA Sequence Motifs Blocking Efficient CRISPR/Cas9-Mediated Gene Editing. *Cell Rep* **26**, 1098-1103 e1093 (2019).
15. Lambert, S.A. et al. The Human Transcription Factors. *Cell* **172**, 650-665 (2018).
16. Uhlen, M. et al. Proteomics. Tissue-based map of the human proteome. *Science* **347**, 1260419 (2015).
17. Braschi, B. et al. Genenames.org: the HGNC and VGNC resources in 2019. *Nucleic Acids Res* **47**, D786-D792 (2019).
18. Smedley, D. et al. BioMart--biological queries made easy. *BMC Genomics* **10**, 22 (2009).
19. Uhlen, M. et al. The human secretome. *Sci Signal* **12** (2019).
20. Abugessaisa, I. et al. FANTOM5 CAGE profiles of human and mouse reprocessed for GRCh38 and GRCm38 genome assemblies. *Sci Data* **4**, 170107 (2017).
21. Frankish, A. et al. GENCODE reference annotation for the human and mouse genomes. *Nucleic Acids Res* **47**, D766-D773 (2019).

Supplementary Figures

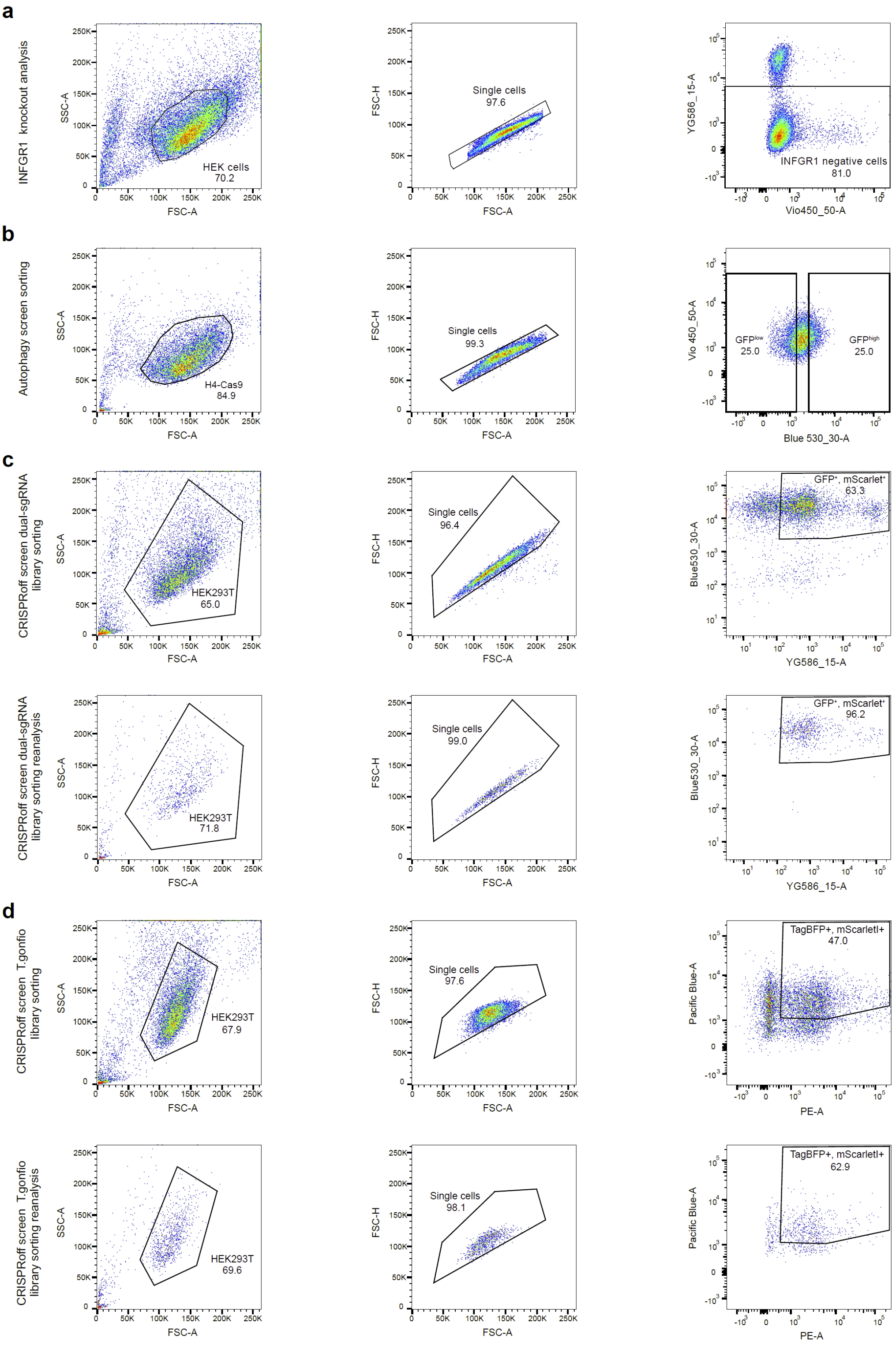

Supplementary Fig.1| Example scatter plots for flow-cytometry-based cell analysis and cell sorting. a, An example of the gating strategy for analysing the efficiency of gene knockout, activation, or epigenetic silencing in HEK293 or HEK293T cells. The plot shows data from *INFGR1* knockout. **b**, An example of the gating strategy for sorting GFP^{high} and GFP^{low} H4-Cas9 cells for the pooled autophagy screens. **c** and **d**, Examples of gating strategies for sorting GFP and mScarletI (**c**) or TagBFP and mScarletI (**d**) double-positive HEK293T cells and the reanalysis of a subsample of the corresponding sorted cells for the essentialome CRISPRoff screens.

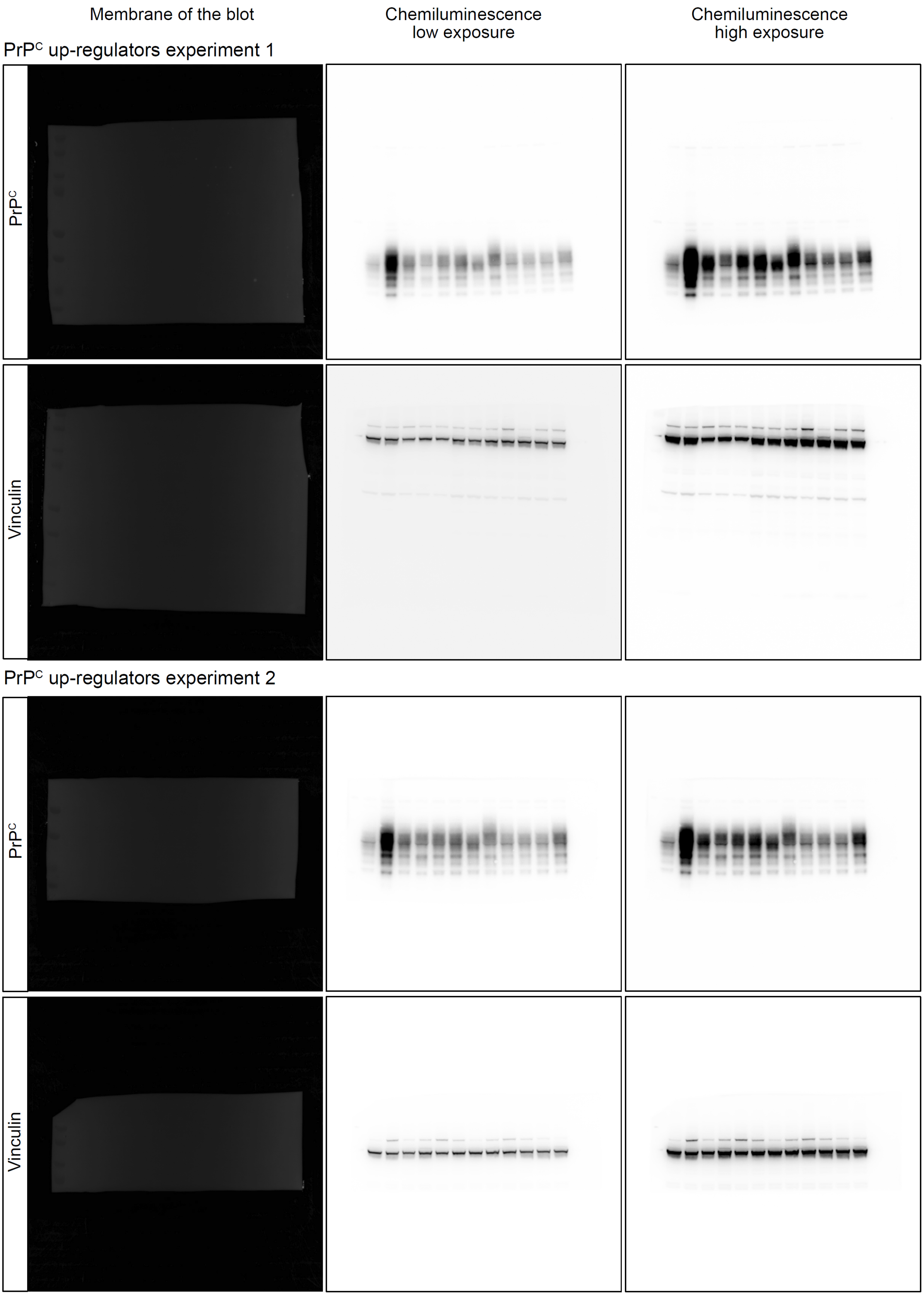

Supplementary Fig.2| Original uncropped gel images of PrP^{C} western blot for up-regulators experiment 1 and 2.

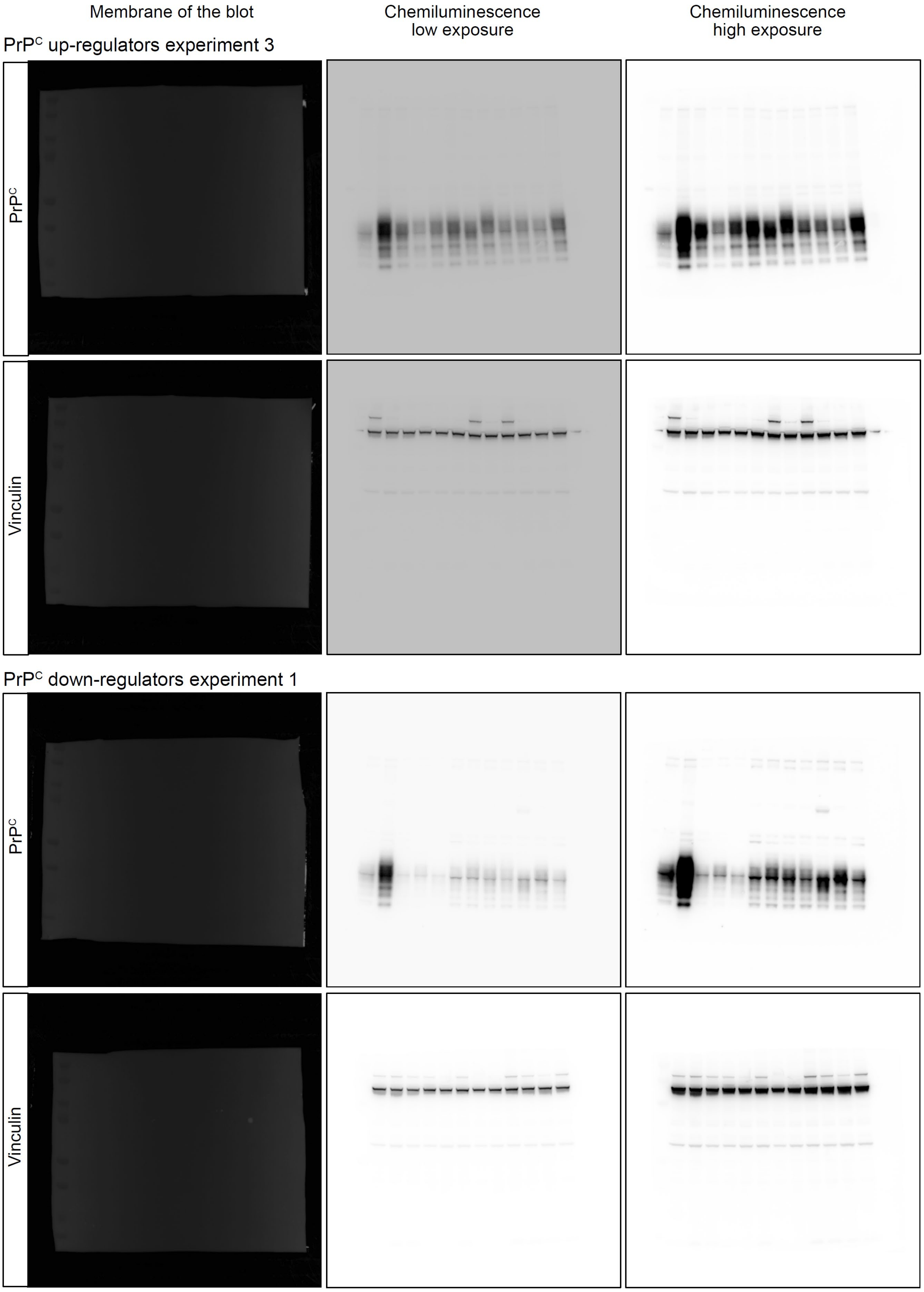

Supplementary Fig.3| Original uncropped gel images of PrP^{C} western blot for up-regulators experiment 3 and down-regulators experiment 1.

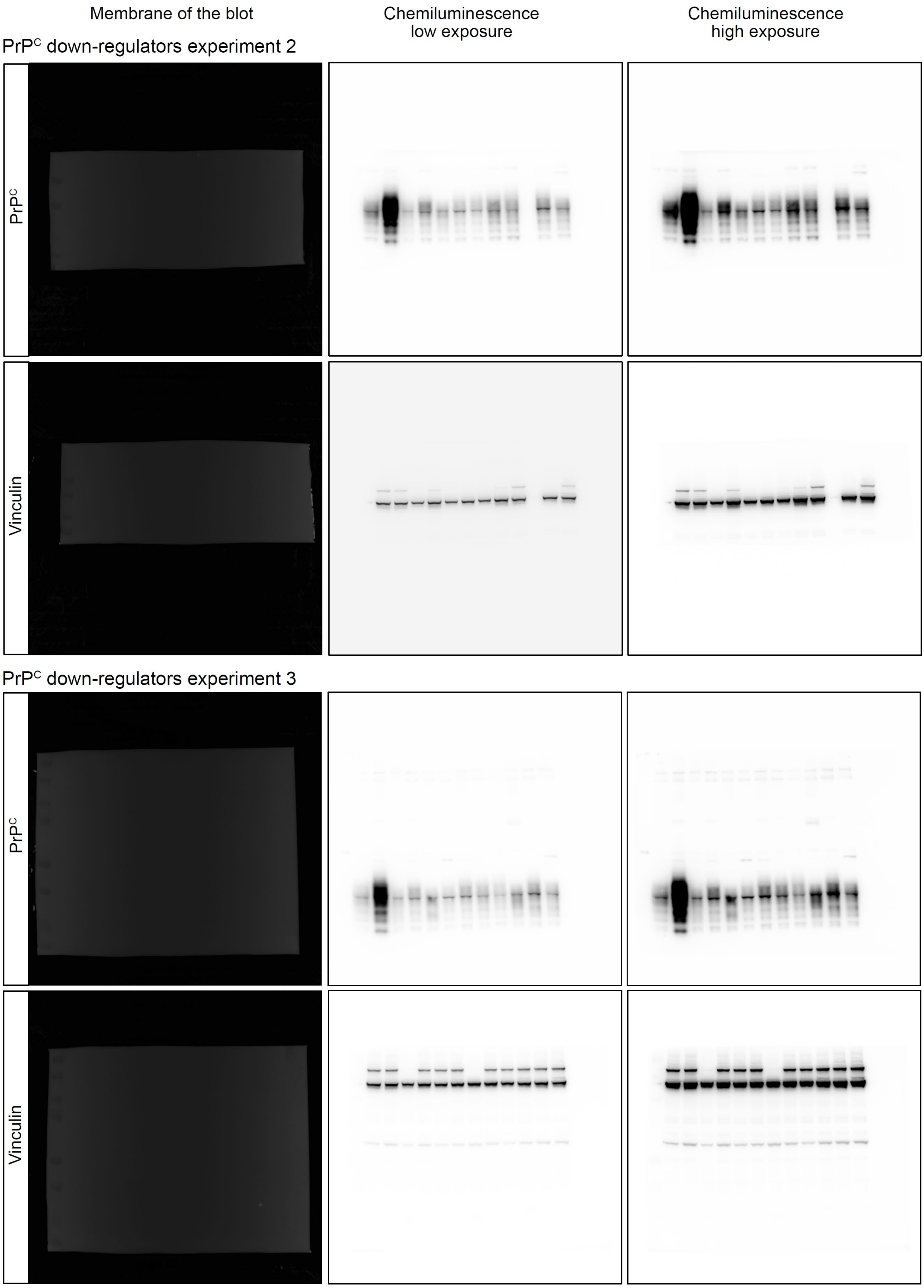

Supplementary Fig.4| Original uncropped gel images of PrP^C western blot for down-regulators experiment 2 and 3.

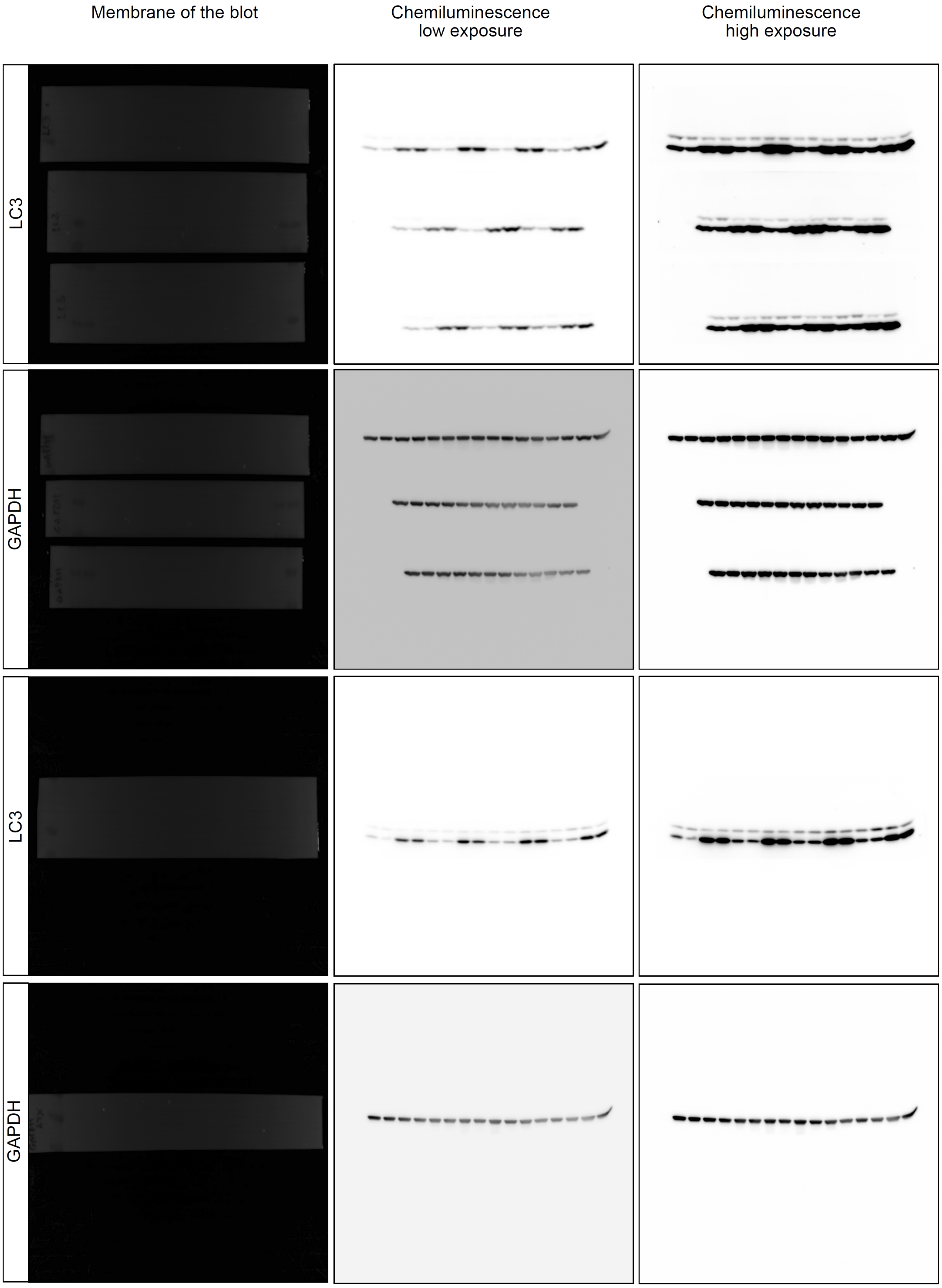

Supplementary Fig.5| Original uncropped gel images of LC3 western blot with two biological repeats.

Supplementary Information

1. **Plasmid map (created with SnapGene) and DNA sequence of the pYJA5 empty vector** (hU6, AmpR, and trcrRNA4 are coloured, for the other features, refer to the annotation of qgRNA-pYJA5).

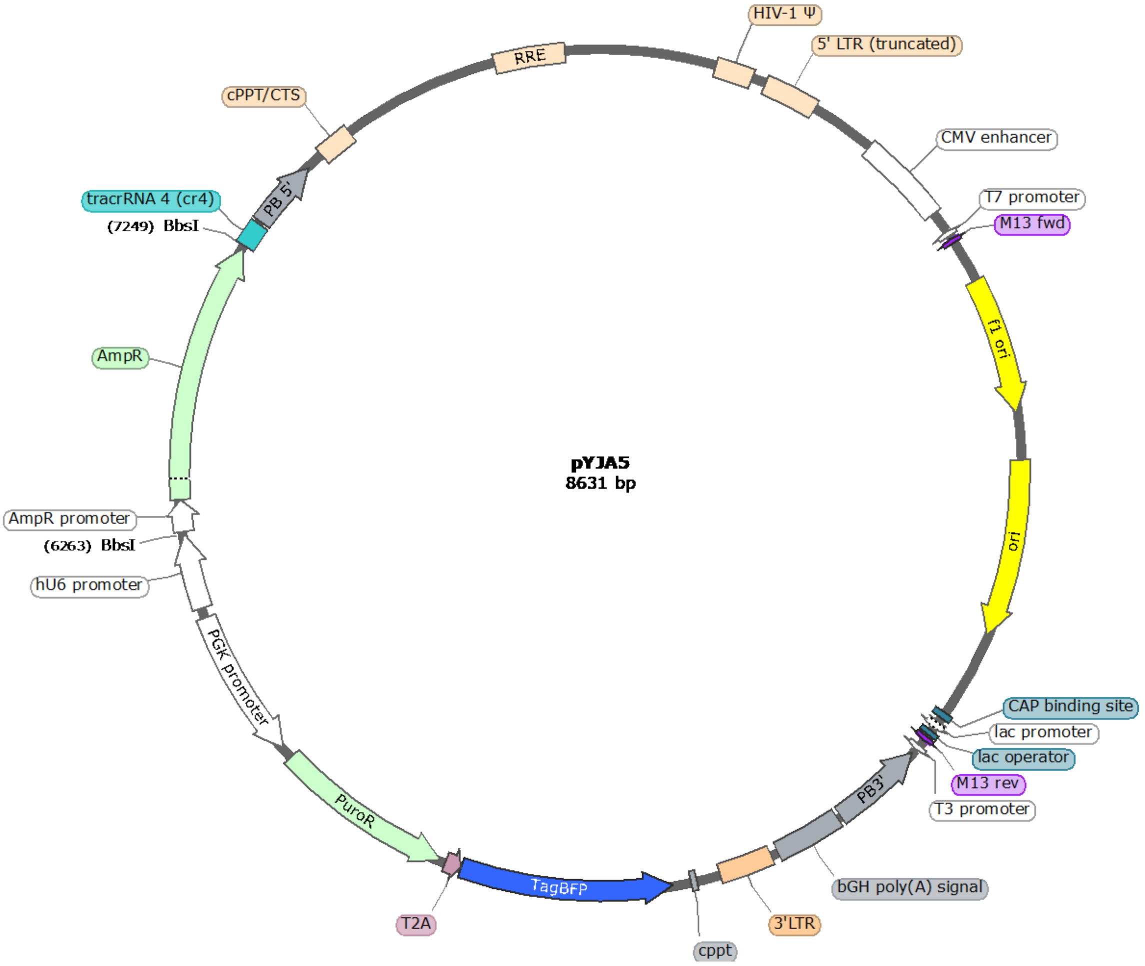

TCCCTCATATCTCCTCCTCCAGGTCTGAAGATCAGCGGCCGGCCGCTTGCTGTGCGGTGGTCTTA
CTTTTGTTTTGCTCTTCCTCTATCTTGTCTAAAGCTTCCTTGGTGTCTTTTATCTCTATCCTTTGATG
CACACAATAGAGGGTTGCTACTGTATTATATAATGATCTAAGTTCTTCTGATCCTGTCTGAAGGGAT
GGTTGTAGCTGTCCCAGTATTTGTCTACAGCCTTCTGATGTTTCTAACAGGCCAGGATTAACTGCG
AATCGTTCTAGCTCCCTGCTTGCCCATACTATATGTTTTAATTTATATTTTTTCTTTCCCCCTGGCCTT
AACCGAATTTTTTCCCATCGCGATCTAATTCTCCCCCGCTTAATACTGACGCTCTCGCACCCATCTC
TCTCCTTCTAGCCTCCGCTAGTCAAAATTTTTGGCGTACTCACCAGTCGCCGCCCCTCGCCTCTT
GCCGTGCGCGCTTCAGCAAGCCGAGTCCTGCGTCGAGAGAGCTCCTCTGGTTTCCCTTTCGCTT
TCAAGTCCCTGTTCGGGCGCCACTGCTAGAGATTTTCCACACTGACTAAAAGGGTCTGAGGGATC
TCTAGTTACCAGAGTCACACAACAGACGGGCACACACTACTTGAAGCACTCAAGGCAAGCTTTAT
TGAGGCTTAAGCAGTGGGTTCCCTAGTTAGCCAGAGAGCTCCCAGGCTCAGATCTGGTCTAACCA
GAGAGACCCAGTACAGGCAAAACGCGCTGCTTATATAGACCTCCCACCGTACACGCCTACCGCCC
ATTTGCGTCAATGGGGCGGAGTTGTTACGACATTTTGGAAAGTCCCGTTGATTTTGGTGCCAAAA
CAAACTCCCATTGACGTCAATGGGGTGGAGACTTGGAAATCCCCGTGAGTCAAACCGCTATCCAC
GCCCATTGATGTACTGCCAAAACCGCATCACCATGGTAATAGCGATGACTAATACGTAGATGTACTG
CCAAGTAGGAAAGTCCCATAAGGTCATGTACTGGGCATAATGCCAGGCGGGCCATTTACCGTCATT
GACGTCAATAGGGGGCGTACTTGGCATATGATACACTTGATGTACTGCCAAGTGGGCAGTTTACC
GTAAATACTCCACCCATTGACGTCAATGGAAAGTCCCTATTGGCGTTACTATGGGAACATACGTCAT
TATTGACGTCAATGGGCGGGGGTCGTTGGGCGGTCAGCCAGGCGGGCCATTTACCGTAAGTTAT
GTAACGCGGAACTCCATATATGGGCTATGAACTAATGACCCCGTAATTGATTACTATTAATAACTAGT

CAATTCGCCCTATAGTGAGTCGTATTACGCGCGCTCACTGGCCGTCGTTTTACAACGTCGTGACT
GGGAAAACCCTGGCGTTACCCAACTTAATCGCCTTGCAGCACATCCCCCTTTCGCCAGCTGGCG
TAATAGCGAAGAGGCCCGCACCGATCGCCCTTCCCAACAGTTGCGCAGCCTGAATGGCGAATGG
GACGCGCCCTGTAGCGGCGCATTAAGCGCGGCGGGTGTGGTGGTTACGCGCAGCGTGACCGCT
ACACTTGCCAGCGCCCTAGCGCCCGCTCCTTTCGCTTTCTTCCCTTCCTTTCTCGCCACGTTCGC
CGGCTTTCCCCGTCAAGCTCTAAATCGGGGGCTCCCTTTAGGGTTCCGATTTAGTGCTTTACGGC
ACCTCGACCCCAAAAAACTTGATTAGGGTGATGGTTCACGTAGTGGGCCATCGCCCTGATAGACG
GTTTTTCGCCCTTTGACGTTGGAGTCCACGTTCTTTAATAGTGGACTCTTGTTCCAAACTGGAACA
ACACTCAACCCTATCTCGGTCTATTCTTTTGATTTATAAGGGATTTTGCCGATTTCGGCCTATTGGTT
AAAAAATGAGCTGATTTAACAAAAATTTAACGCGAATTTTAACAAAATATTAACGCTTACAATTTAGG
TGGCACTTTTCGGGGAAATGTGCGCGGAACCCCTATTTGTTTATTTTTCTAAATACATTCAAATATGT
ATCCGCTCATGACCAAAATCCCTTAACGTGAGTTTTCGTTCCACTGAGCGTCAGACCCCGTAGAA
AAGATCAAAGGATCTTCTTGAGATCCTTTTTTTCTGCGCGTAATCTGCTGCTTGCAAACAAAAAAAC
CACCGCTACCAGCGGTGGTTTGTTTGCCGGATCAAGAGCTACCAACTCTTTTTCCGAAGGTAACT
GGCTTCAGCAGAGCGCAGATACCAAATACTGTtCTTCTAGTGTAGCCGTAGTTAGGCCACCACTTC
AAGAACTCTGTAGCACCGCCTACATACCTCGCTCTGCTAATCCTGTTACCAGTGGCTGCTGCCAG
TGGCGATAAGTCGTGTCTTACCGGGTTGGACTCAAGACGATAGTTACCGGATAAGGCGCAGCGGT
CGGGCTGAACGGGGGGTTCGTGCACACAGCCCAGCTTGGAGCGAACGACCTACACCGAACTGA
GATACCTACAGCGTGAGCTATGAGAAAGCGCCACGCTTCCCGAAGGGAGAAAGGCGGACAGGTA
TCCGGTAAGCGGCAGGGTCGGAACAGGAGAGCGCACGAGGGAGCTTCCAGGGGGAAACGCCT
GGTATCTTTATAGTCCTGTCGGGTTTCGCCACCTCTGACTTGAGCGTCGATTTTTGTGATGCTCGT
CAGGGGGGCGGAGCCTATGGAAAAACGCCAGCAACGCGGCCTTTTTACGGTTCCTGGCCTTTTG
CTGGCCTTTTGCTCACATGTTCTTTCCTGCGTTATCCCCTGATTCTGTGGATAACCGTATTACCGCC
TTTGAGTGAGCTGATACCGCTCGCCGCAGCCGAACGACCGAGCGCAGCGAGTCAGTGAGCGAG
GAAGCGGAAGAGCGCCCAATACGCAAACCGCCTCTCCCCGCGCGTTGGCCGATTCATTAATGCA
GCTGGCACGACAGGTTTCCCGACTGGAAAGCGGGCAGTGAGCGCAACGCAATTAATGTGAGTTA
GCTCACTCATTAGGCACCCCAGGCTTTACACTTTATGCTcCCGGCTCGTATGTTGTGTGGAATTGT
GAGCGGATAACAATTTCACACAGGAAACAGCTATGACCATGATTACGCCAAGCGCGCAATTAACCC
TCACTAAAGGGAACAAAAGCTTAATTTAACCCTAGAAAGATAGTCTGCGTAAAATTGACGCATGCAT
TCTTGAAATATTGCTCTCTCTTTCTAAATAGCGCGAATCCGTCGCTGTGCATTTAGGACATCTCAGT
CGCCGCTTGGAGCTCCCGTGAGGCGTGCTTGTCAATGCGGTAAGTGTCACTGATTTTGAACTATA
ACGACCGCGTGAGTCAAAATGACGCATGATTATCTTTTACGTGACTTTTAAGATTTAACTCATACGA
TAATTATATTGTTATTTCATGTTCTACTTACGTGATAACTTATTATATATATATTTTCTTGTTATAGATATC
ATTAACGGCCGCCCATAGAGCCCACCGCATCCCCAGCATGCCTGCTATTGACTTCCCAATCCTCC
CCCTTGCTGTCCTGCCCCACCCCACCCCCCAGAATAGAATGACACCTACTCAGACAATGCGATGC
AATTTCCTCATTTTATTAGGAAAGGACAGTGGGAGTGGCACCTTCCAGGGTCAAGGAAGGCACGG
GGGAGGGGCAAACAACAGATGGCTGGCAACTAGAAGGCACAGTCGAGGCTGATCAGCGGGTTT
AAACTGCTAGAGATTTTCCACACTGACTAAAAGGGTCTGAGGGATCTCTAGTTACCAGAGTCACAC
AACAGACGGGCACACACTACTTGAAGCACTCAAGGCAAGCTTTATTGAGGCTTAAGCAGTGGGTT
CCCTAGTTAGCCAGAGAGCTCCCAGGCTCAGATCTGGTCTAACCAGAGAGACTCTGGTCTAACCA
GAGAGACCCAGTACAGGCAAAAAGCAGATCTTGACTTCTTTGGGAGTGAATTAGCCCTTCCAGTC
CCCCCTTTTCTTTTAAAAAGTGGCTAAGATCTACAGCTGCCTTGTAAGTCATTGGTCTTAAAGGTAC
CTAGCGGCCGCTCAATTAAGCTTGTGCCCCAGTTTGCTAGGGAGGTCGCAGTATCTGGCCACTG
CCACCTCGTGCTGCTCGACGTAGGTCTCGTTGTTGGCCTCCTTGATTCTTTCCAGTCTGTAGTCC
ACATAGTAGACGCCAGGCATCTTGAGGTTCTTAGCGGGTTTCTTGGATCTATATGTGGTCTTGATG
TTTGCGATCAGATGGCTCCCGCCCACGAGCTTCAGGGCCATGTCGTTTCTGCCTTCCAGGCCGC
CGTCAGCGGGGTACAGaGTCTCGGTGAAGGCCTCCCAGCCGAGTGTTTTCTTCTGCATCACAGG
GCCGTTGGATGTGAAGTTCACCCCTCTGATCTTGACGTTGTAGATGAGGCAGCCGTCCTGGAGG
CTGGTGTCCTGGGTAGCGGTCAGCACGCCCCCGTCCTCGTATGTGGTGACTCTCTCCCATGTGA
AGCCCTCAGGGAAGGACTGCTTGAAGAAGTCGGGGATGCCCTGGGTGTGGTTGATGAAGGTCTT
GCTGCCGTAGAGGAAGCTAGTAGCCAGGATGTCGAAGGCGAAGGGGAGAGGGCCGCCCTCGAC
CACCTTGATTCTCATGGTCTGGGTGCCCTCGTAGGGCTTGCCTTCGCCCTCGGATGTGCACTTGA
AGTGATGGTTGTCCACGGTGCCCTCCATGTACAGCTTCATGTGCATGTTCTCCTTAATCAGCTCGC
TCATTGGGCCAGGATTCTCCTCCACGTCACCGCATGTTAGGAGACTTCCTCTGCCCTCTCCTCCG
GACCCGCCGCCGGCACCGGGCTTGCGGGTCATGCACCAGGTGCGCGGTCCTTCGGGCACCTC
GACGTCGGCGGTGACGGTGAAGCCGAGCCGCTCGTAGAAGGGGAGGTTGCGGGGCGCGGAG

GTCTCCAGGAAGGCGGGCACCCCGGCGCGCTCGGCCGCCTCCACTCCGGGGAGCACGACGGC
GCTGCCCAGACCCTTGCCCTGGTGGTCGGGCGAGACcCCGACGGTGGCCAGGAACCACGCGG
GCTCCTTGGGCCGGTGCGGCGCCAGGAGGCCTTCCATCTGTTGCTGCGCGGCCAGCCGGGAA
CCGCTCAACTCGGCCATGCGCGGGCCGATCTCGGCGAACACCGCCCCCGCTTCGACGCTCTCC
GGCGTGGTCCAGACCGCCACCGCGGCGCCGTCGTCCGCGACCCACACCTTGCCGATGTCGAG
CCCGACGCGCGTGAGGAAGAGTTCTTGCAGCTCGGTGACCCGCTCGATGTGGCGGTCCGGATC
GACGGTGTGGCGCGTGGCGGGGTAGTCGGCGAACGCGGCGGCGAGGGTGCGTACGGCCCTG
GGGACGTCGTCGCGGGTGGCGAGGCGCACCGTGGGCTTGTACTCGGTCATGGTAAGCTTCAGC
TGCTCGAGATCTAGATGGATGCAGGTCGAAAGGCCCGGAGATGAGGAAGAGGAGAACAGCGCG
GCAGACGTGCGCTTTTGAAGCGTGCAGAATGCCGGGCCTCCGGAGGACCTTCGGGCGCCCGCC
CCGCCCCTGAGCCCGCCCCTGAGCCCGCCCCCGGACCCACCCCTTCCCAGCCTCTGAGCCCA
GAAAGCGAAGGAGCAAAGCTGCTATTGGCCGCTGCCCCAAAGGCCTACCCGCTTCCATTGCTCA
GCGGTGCTGTCCATCTGCACGAGACTAGTGAGACTGCTACTTCCATTTGTCACGTCCTGCACGAC
GCGAGCTGCGGGGCGGGGGGGAACTTCCTGACTAGGGGAGGAGTAGAAGGTGGCGCGAAGGG
GCCACCAAAGAACGGAGCCGGTTGGCGCCTACCGGTGGATGTGGAATGTGTGCGAGGCCAGAG
GCCACTTGTGTAGCGCCAAGTGCCCAGCGGGGCTGCTAAAGCGCATGCTCCAGACTGCCTTGG
GAAAAGCGCCTCCCCTACCCGGTAGAATTGGATCcCCAAGGTCGGGCAGGAAgagggcctatttcccatg
attccttcatatttgcatatacgatacaaggctgttagagagataattagaattaatttgactgtaaacacaaagatattagtacaaaatacgtgac
gtagaaagtaataatttcttgggtagtttgcagttttaaaattatgttttaaaatggactatcatatgcttaccgtaacttgaaagtatttcgatttcttggc
tttatatatcttgtggaaaggacgaaacaccGggGTCTTCCGCGGAACCCCTATTTGTTTATTTTTCTAAATACATT
CAAATATGTATCCGCTCATGAGACAATAACCCTGATAAATGCTTCAATAATATTGAAAAAGGAAGAGT
ATGAGTATTCAACATTTCCGTGTCGCCCTTATTCCCTTTTTTGCGGCATTTTGCCTTCCTGTTTTTG
CTCACCCAGAAACGCTGGTGAAAGTAAAAGATGCTGAAGATCAGTTGGGTGCACGAGTGGGTTA
CATCGAACTGGATCTCAACAGCGGTAAGATCCTTGAGAGTTTTCGCCCCGAAGAACGTTTTCCAA
TGATGAGCACTTTTAAAGTTCTGCTATGTGGCGCGGTATTATCCCGTATTGACGCCGGGCAAGAGC
AACTCGGTCGCCGCATACACTATTCTCAGAATGACTTGGTTGAGTACTCACCAGTCACAGAAAAG
CATCTTACGGATGGCATGACAGTAAGAGAATTATGCAGTGCTGCCATAACCATGAGTGATAACACT
GCGGCCAACTTACTTCTGACAACGATCGGAGGACCGAAGGAGCTAACCGCTTTTTTGCACAACAT
GGGGGATCATGTAACTCGCCTTGATCGTTGGGAACCGGAGCTGAATGAAGCCATACCAAACGAC
GAGCGTGACACCACGATGCCTGTAGCAATGGCAACAACGTTGCGCAAACTATTAACTGGCGAACT
ACTTACTCTAGCTTCCCGGCAACAATTAATAGACTGGATGGAGGCGGATAAAGTTGCAGGACCACT
TCTGCGCTCGGCCCTTCCGGCTGGCTGGTTTATTGCTGATAAATCTGGAGCCGGTGAGCGTGGG
TCTCGCGGTATCATTGCAGCACTGGGGCCAGATGGTAAGCCCTCCCGTATCGTAGTTATCTACAC
GACGGGGAGTCAGGCAACTATGGATGAACGAAATAGACAGATCGCTGAGATAGGTGCCTCACTG
ATTAAGCATTGGTAAGAAGACggGTTTCAGAGCTACAGCAGAAATGCTGTAGCAAGTTGAAATAAG
GCTAGTCCGTTATCAACTTGAAAAAGTGGCACCGAGTCGGTGCttttttGCTTTGTTACTTTATAGAAG
AAATTTTGAGTTTTTGTTTTTTTTTAATAAATAAATAAACATAAATAAATTGTTTGTTGAATTTATTATTA
GTATGTAAGTGTAAATATAATAAAACTTAATATCTATTCAAATTAATAAATAAACCTCGATATACAGACC
GATAAAACACATGCGTCAATTTTACGCATGATTATCTTTAACGTACGTCACAATATGATTATCTTTCTA
GGGTTAAGTAACGCGTAGGGCCCGGTACTAACCAAACTGGATCTCTGCTGTCCCTGTAATAAACC
CGAAAATTTTGAATTTTTGTAATTTGTTTTTGTAATTCTTTAGTTTGTATGTCTGTTGCTATTATGTCTA
CTATTCTTTCCCCTGCACTGTACCCCCCAATCCCCCCTTTTCTTTTAAAATTGTGGATGAATACTGC
CATTTGTCTGCAGAATTGGCGCACGCAGTGCCGATCCGTTCACTAATCGAATGGATCTGTCTCTGT
CTCTCTCTCCACCTTCTTCTTCTATTCCTTCGGGCCTGTCGGGTCCCCTCGGGGTTGGGAGGTG
GGTCTGAAACGATAATGGTGAATATCCCTGCCTAACTCTATTCACTATAGAAAGTACAGCAAAAACT
ATTCTTAAACCTACCAAGCCTCCTACTATCATTATGAATAATTTTATATACCACAGCCAATTTGTTATG
TTAAACCAATTCCACAAACTTGCCCATTTATCTAATTCCAATAATTCTTGTTCATTCTTTTCTTGCTGG
TTTTGCGATTCTTCAATTAAGGAGTGTATTAAGCTTGTGTAATTGTTAATTTCTCTGTCCCACTCCAT
CCAGGTCGTGTGATTCCAAATCTGTTCCAGAGATTTATTACTCCAACTAGCATTCCAAGGCACAGC
AGTGGTGCAAATGAGTTTTCCAGAGCAACCCCAAATCCCCAGGAGCTGTTGATCCTTTAGGTATCT
TTCCACAGCCAGGATTCTTGCCTGGAGCTGCTTGATGCCCCAGACTGTGAGTTGCAACAGATGCT
GTTGCGCCTCAATAGCCCTCAGCAAATTGTTCTGCTGCTGCACTATACCAGACAATAATTGTCTGG
CCTGTACCGTCAGCGTCATTGACGCTGCGCCCATAGTGCTTCCTGCTGCTCCCAAGAACCCAAG
GAACAAAGCTCCTATTCCCACTGCTCTTTTTTCTCTCTGCACCACTCTTCTCTTTGCCTTGGTGGG
TGCTACTCCTAATGGTTCAATTTTTACTACTTTATATTTATATAATTCACTTCTCCAATTG

2. **Plasmid map (created with SnapGene) and DNA sequence of the qgRNA-pYJA5 vector** (N_{20} indicates sgRNA sequence, all the qgRNA features annotated)

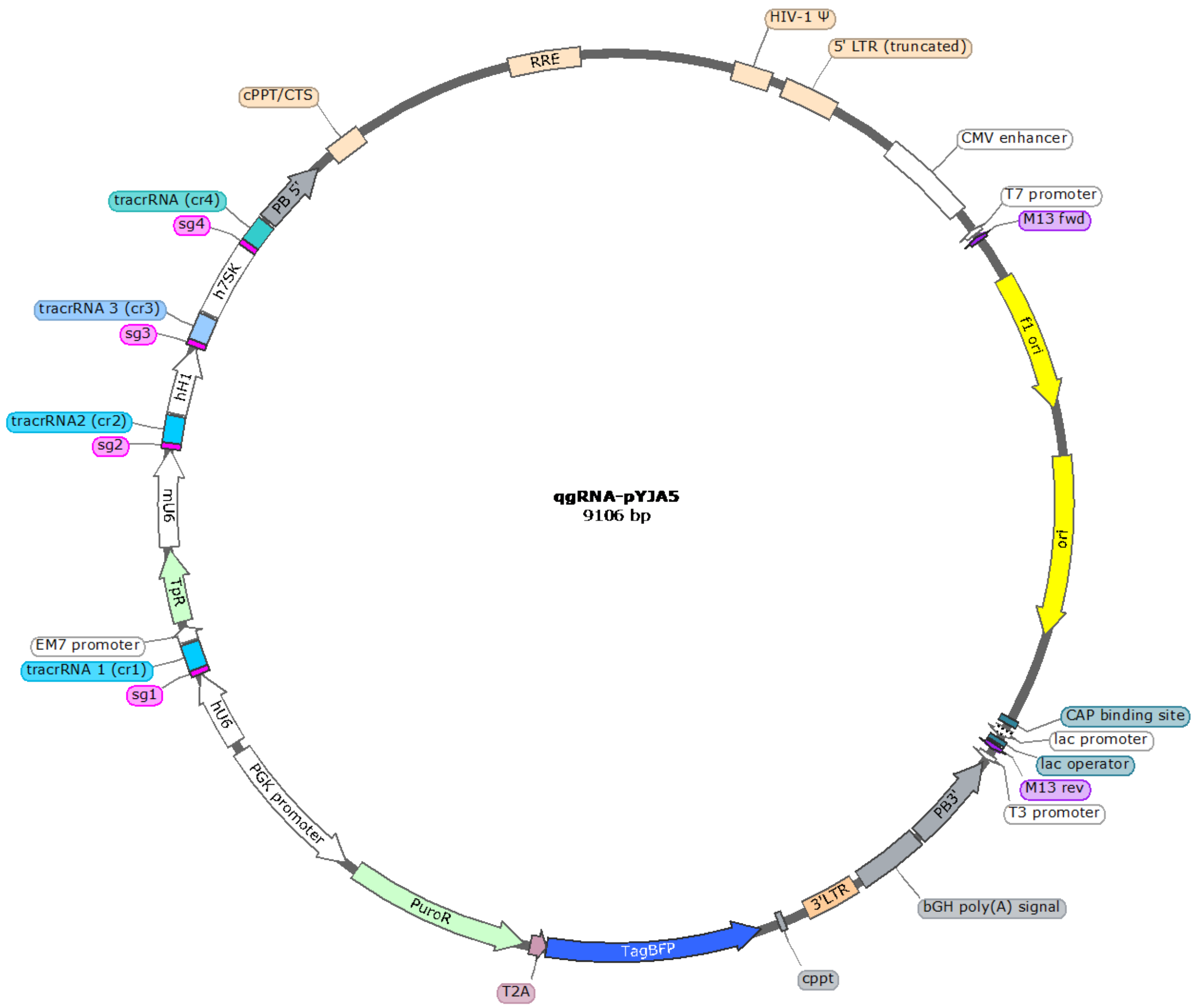

TCCCTCATATCTCCTCCTCCAGGTCTGAAGATCAGCGGCCGGCCGCTTGCTGTGCGGTGGTCTTA
CTTTTGTTTTGCTCTTCCTCTATCTTGTCTAAAGCTTCCTTGGTGTCTTTTATCTCTATCCTTTGATG
CACACAATAGAGGGTTGCTACTGTATTATATAATGATCTAAGTTCTTCTGATCCTGTCTGAAGGGAT
GGTTGTAGCTGTCCCAGTATTTGTCTACAGCCTTCTGATGTTTCTAACAGGCCAGGATTAACTGCG
AATCGTTCTAGCTCCCTGCTTGCCCATACTATATGTTTTAATTTATATTTTTTCTTTCCCCCTGGCCTT
AACCGAATTTTTTCCCATCGCGATCTAATTCTCCCCCGCTTAATACTGACGCTCTCGCACCCATCTC
TCTCCTTCTAGCCTCCGCTAGTCAAAATTTTTGGCGTACTCACCAGTCGCCGCCCCTCGCCTCTT
GCCGTGCGCGCTTCAGCAAGCCGAGTCCTGCGTCGAGAGAGCTCCTCTGGTTTCCCTTTCGCTT
TCAAGTCCCTGTTCGGGCGCCACTGCTAGAGATTTTCCACACTGACTAAAAGGGTCTGAGGGATC
TCTAGTTACCAGAGTCACACAACAGACGGGCACACACTACTTGAAGCACTCAAGGCAAGCTTTAT
TGAGGCTTAAGCAGTGGGTTCCCTAGTTAGCCAGAGAGCTCCCAGGCTCAGATCTGGTCTAACCA
GAGAGACCCAGTACAGGCAAAACGCGCTGCTTATATAGACCTCCCACCGTACACGCCTACCGCCC
ATTTGCGTCAATGGGGCGGAGTTGTTACGACATTTTGGAAAGTCCCGTTGATTTTGGTGCCAAAA
CAAACTCCCATTGACGTCAATGGGGTGGAGACTTGGAAATCCCCGTGAGTCAAACCGCTATCCAC
GCCCATTGATGTACTGCCAAAACCGCATCACCATGGTAATAGCGATGACTAATACGTAGATGTACTG
CCAAGTAGGAAAGTCCCATAAGGTCATGTACTGGGCATAATGCCAGGCGGGCCATTTACCGTCATT
GACGTCAATAGGGGGCGTACTTGGCATATGATACACTTGATGTACTGCCAAGTGGGCAGTTTACC
GTAAATACTCCACCCATTGACGTCAATGGAAAGTCCCTATTGGCGTTACTATGGGAACATACGTCAT
TATTGACGTCAATGGGCGGGGGTCGTTGGGCGGTCAGCCAGGCGGGCCATTTACCGTAAGTTAT
GTAACGCGGAACTCCATATATGGGCTATGAACTAATGACCCCGTAATTGATTACTATTAATAACTAGT
CAATTCGCCCTATAGTGAGTCGTATTACGCGCGCTCACTGGCCGTCGTTTTACAACGTCGTGACT

GGGAAAACCCTGGCGTTACCCAACTTAATCGCCTTGCAGCACATCCCCCTTTCGCCAGCTGGCG
TAATAGCGAAGAGGCCCGCACCGATCGCCCTTCCCAACAGTTGCGCAGCCTGAATGGCGAATGG
GACGCGCCCTGTAGCGGCGCATTAAGCGCGGCGGGTGTGGTGGTTACGCGCAGCGTGACCGCT
ACACTTGCCAGCGCCCTAGCGCCCGCTCCTTTCGCTTTCTTCCCTTCCTTTCTCGCCACGTTCGC
CGGCTTTCCCCGTCAAGCTCTAAATCGGGGGCTCCCTTTAGGGTTCCGATTTAGTGCTTTACGGC
ACCTCGACCCCAAAAAACTTGATTAGGGTGATGGTTCACGTAGTGGGCCATCGCCCTGATAGACG
GTTTTTCGCCCTTTGACGTTGGAGTCCACGTTCTTTAATAGTGGACTCTTGTTCCAAACTGGAACA
ACACTCAACCCTATCTCGGTCTATTCTTTTGATTTATAAGGGATTTTGCCGATTTCGGCCTATTGGTT
AAAAAATGAGCTGATTTAACAAAAATTTAACGCGAATTTTAACAAAATATTAACGCTTACAATTTAGG
TGGCACTTTTCGGGGAAATGTGCGCGGAACCCCTATTTGTTTATTTTTCTAAATACATTCAAATATGT
ATCCGCTCATGACCAAAATCCCTTAACGTGAGTTTTCGTTCCACTGAGCGTCAGACCCCGTAGAA
AAGATCAAAGGATCTTCTTGAGATCCTTTTTTTCTGCGCGTAATCTGCTGCTTGCAAACAAAAAAAC
CACCGCTACCAGCGGTGGTTTGTTTGCCGGATCAAGAGCTACCAACTCTTTTTCCGAAGGTAACT
GGCTTCAGCAGAGCGCAGATACCAAATACTGTtCTTCTAGTGTAGCCGTAGTTAGGCCACCACTTC
AAGAACTCTGTAGCACCGCCTACATACCTCGCTCTGCTAATCCTGTTACCAGTGGCTGCTGCCAG
TGGCGATAAGTCGTGTCTTACCGGGTTGGACTCAAGACGATAGTTACCGGATAAGGCGCAGCGGT
CGGGCTGAACGGGGGGTTCGTGCACACAGCCCAGCTTGGAGCGAACGACCTACACCGAACTGA
GATACCTACAGCGTGAGCTATGAGAAAGCGCCACGCTTCCCGAAGGGAGAAAGGCGGACAGGTA
TCCGGTAAGCGGCAGGGTCGGAACAGGAGAGCGCACGAGGGAGCTTCCAGGGGGAAACGCCT
GGTATCTTTATAGTCCTGTCGGGTTTCGCCACCTCTGACTTGAGCGTCGATTTTTGTGATGCTCGT
CAGGGGGGCGGAGCCTATGGAAAAACGCCAGCAACGCGGCCTTTTTACGGTTCCTGGCCTTTTG
CTGGCCTTTTGCTCACATGTTCTTTCCTGCGTTATCCCCTGATTCTGTGGATAACCGTATTACCGCC
TTTGAGTGAGCTGATACCGCTCGCCGCAGCCGAACGACCGAGCGCAGCGAGTCAGTGAGCGAG
GAAGCGGAAGAGCGCCCAATACGCAAACCGCCTCTCCCCGCGCGTTGGCCGATTCATTAATGCA
GCTGGCACGACAGGTTTCCCGACTGGAAAGCGGGCAGTGAGCGCAACGCAATTAATGTGAGTTA
GCTCACTCATTAGGCACCCCAGGCTTTACACTTTATGCTcCCGGCTCGTATGTTGTGTGGAATTGT
GAGCGGATAACAATTTCACACAGGAAACAGCTATGACCATGATTACGCCAAGCGCGCAATTAACCC
TCACTAAAGGGAACAAAAGCTTAATTTAACCCTAGAAAGATAGTCTGCGTAAAATTGACGCATGCAT
TCTTGAAATATTGCTCTCTCTTTCTAAATAGCGCGAATCCGTCGCTGTGCATTTAGGACATCTCAGT
CGCCGCTTGGAGCTCCCGTGAGGCGTGCTTGTCAATGCGGTAAGTGTCACTGATTTTGAACTATA
ACGACCGCGTGAGTCAAAATGACGCATGATTATCTTTTACGTGACTTTTAAGATTTAACTCATACGA
TAATTATATTGTTATTTCATGTTCTACTTACGTGATAACTTATTATATATATATTTTCTTGTTATAGATATC
ATTAACGGCCGCCCATAGAGCCCACCGCATCCCCAGCATGCCTGCTATTGACTTCCCAATCCTCC
CCCTTGCTGTCCTGCCCCACCCCACCCCCCAGAATAGAATGACACCTACTCAGACAATGCGATGC
AATTTCCTCATTTTATTAGGAAAGGACAGTGGGAGTGGCACCTTCCAGGGTCAAGGAAGGCACGG
GGGAGGGGCAAACAACAGATGGCTGGCAACTAGAAGGCACAGTCGAGGCTGATCAGCGGGTTT
AAACTGCTAGAGATTTTCCACACTGACTAAAAGGGTCTGAGGGATCTCTAGTTACCAGAGTCACAC
AACAGACGGGCACACACTACTTGAAGCACTCAAGGCAAGCTTTATTGAGGCTTAAGCAGTGGGTT
CCCTAGTTAGCCAGAGAGCTCCCAGGCTCAGATCTGGTCTAACCAGAGAGACTCTGGTCTAACCA
GAGAGACCCAGTACAGGCAAAAAGCAGATCTTGACTTCTTTGGGAGTGAATTAGCCCTTCCAGTC
CCCCCTTTTCTTTTAAAAAGTGGCTAAGATCTACAGCTGCCTTGTAAGTCATTGGTCTTAAAGGTAC
CTAGCGGCCGCTCAATTAAGCTTGTGCCCCAGTTTGCTAGGGAGGTCGCAGTATCTGGCCACTG
CCACCTCGTGCTGCTCGACGTAGGTCTCGTTGTTGGCCTCCTTGATTCTTTCCAGTCTGTAGTCC
ACATAGTAGACGCCAGGCATCTTGAGGTTCTTAGCGGGTTTCTTGGATCTATATGTGGTCTTGATG
TTTGCGATCAGATGGCTCCCGCCCACGAGCTTCAGGGCCATGTCGTTTCTGCCTTCCAGGCCGC
CGTCAGCGGGGTACAGaGTCTCGGTGAAGGCCTCCCAGCCGAGTGTTTTCTTCTGCATCACAGG
GCCGTTGGATGTGAAGTTCACCCCTCTGATCTTGACGTTGTAGATGAGGCAGCCGTCCTGGAGG
CTGGTGTCCTGGGTAGCGGTCAGCACGCCCCCGTCCTCGTATGTGGTGACTCTCTCCCATGTGA
AGCCCTCAGGGAAGGACTGCTTGAAGAAGTCGGGGATGCCCTGGGTGTGGTTGATGAAGGTCTT
GCTGCCGTAGAGGAAGCTAGTAGCCAGGATGTCGAAGGCGAAGGGGAGAGGGCCGCCCTCGAC
CACCTTGATTCTCATGGTCTGGGTGCCCTCGTAGGGCTTGCCTTCGCCCTCGGATGTGCACTTGA
AGTGATGGTTGTCCACGGTGCCCTCCATGTACAGCTTCATGTGCATGTTCTCCTTAATCAGCTCGC
TCATTGGGCCAGGATTCTCCTCCACGTCACCGCATGTTAGGAGACTTCCTCTGCCCTCTCCTCCG
GACCCGCCGCCGGCACCGGGCTTGCGGGTCATGCACCAGGTGCGCGGTCCTTCGGGCACCTC
GACGTCGGCGGTGACGGTGAAGCCGAGCCGCTCGTAGAAGGGGAGGTTGCGGGGCGCGGAG
GTCTCCAGGAAGGCGGGCACCCCGGCGCGCTCGGCCGCCTCCACTCCGGGGAGCACGACGGC

GCTGCCCAGACCCTTGCCCTGGTGGTCGGGCGAGACcCCGACGGTGGCCAGGAACCACGCGG
GCTCCTTGGGCCGGTGCGGCGCCAGGAGGCCTTCCATCTGTTGCTGCGCGGCCAGCCGGGAA
CCGCTCAACTCGGCCATGCGCGGGCCGATCTCGGCGAACACCGCCCCCGCTTCGACGCTCTCC
GGCGTGGTCCAGACCGCCACCGCGGCGCCGTCGTCCGCGACCCACACCTTGCCGATGTCGAG
CCCGACGCGCGTGAGGAAGAGTTCTTGCAGCTCGGTGACCCGCTCGATGTGGCGGTCCGGATC
GACGGTGTGGCGCGTGGCGGGGTAGTCGGCGAACGCGGCGGCGAGGGTGCGTACGGCCCTG
GGGACGTCGTCGCGGGTGGCGAGGCGCACCGTGGGCTTGTACTCGGTCATGGTAAGCTTCAGC
TGCTCGAGATCTAGATGGATGCAGGTCGAAAGGCCCGGAGATGAGGAAGAGGAGAACAGCGCG
GCAGACGTGCGCTTTTGAAGCGTGCAGAATGCCGGGCCTCCGGAGGACCTTCGGGCGCCCGCC
CCGCCCCTGAGCCCGCCCCTGAGCCCGCCCCCGGACCCACCCCTTCCCAGCCTCTGAGCCCA
GAAAGCGAAGGAGCAAAGCTGCTATTGGCCGCTGCCCCAAAGGCCTACCCGCTTCCATTGCTCA
GCGGTGCTGTCCATCTGCACGAGACTAGTGAGACTGCTACTTCCATTTGTCACGTCCTGCACGAC
GCGAGCTGCGGGGCGGGGGGAACTTCCTGACTAGGGGAGGAGTAGAAGGTGGCGCGAAGGG
GCCACCAAAGAACGGAGCCGGTTGGCGCCTACCGGTGGATGTGGAATGTGTGCGAGGCCAGAG
GCCACTTGTGTAGCGCCAAGTGCCCAGCGGGGCTGCTAAAGCGCATGCTCCAGACTGCCTTGG
GAAAAGCGCCTCCCCTACCCGGTAGAATTGGATCcCCAAGGTCGGGCAGGAAgagggcctatttcccatg
attccttcatatttgcatatacgatacaaggctgttagagagataattagaattaatttgactgtaaacacaaagatattagtacaaaatacgtgac
gtagaaagtaataatttcttgggtagtttgcagttttaaaattatgttttaaaatggactatcatatgcttaccgtaacttgaaagtatttcgatttcttggc
tttatatatcttgtggaaaggacgaaacaccGNNNNNNNNNNNNNNNNNNNNGTTTAAGAGCTAAGCTGGAAAC
AGCATAGCAAGTTTAAATAAGGCTAGTCCGTTATCAACTTGAAAAAGTGGCACCGAGTCGGTGCttttt
ttgttgacaattaatcatcggcatagtatatcggcatagtataatacgacaaggtgaggaactaaaccatgggtcaaagtagcgatgaagcca
acgctcccgttgcagggcagtttgcgcttcccctgagtgccacctttggcttaggggatcgcgtacgcaagaaatctggtgccgcttggcagggt
caagtcgtcggttggtattgcacaaaactcactcctgaaggctatgcggtcgagtccgaatcccacccaggctcagtgcaaatttatcctgtggc
tgcacttgaacgtgtggcctaatttttttgatccgacgccgccatctctaggcccgcgccggccccctcgcacagacttgtgggagaagctcggc
tactcccctgccccggttaatttgcatataatatttcctagtaactatagaggcttaatgtgcgataaaagacagataatctgttctttttaatactagct
acattttacatgataggcttggatttctataagagatacaaatactaaattattattttaaaaaacagcacaaaaggaaactcaccctaactgtaa
agtaattgtgtgttttgagactataaatatcccttggagaaaagccttgtttGNNNNNNNNNNNNNNNNNNNNGTTTGAGAG
CTAAGCAGAAAGCTGCATAGCAAGTTCAAATAAGGCTAGTCCGTACACAACTTGAAAAAGTGGCA
GCCGAGTCGGCTGCttttttgaacgctgacgtcatcaacccgctccaaggaatcgcgggcccagtgtcactaggcgggaacaccc
agcgcgcgtgcgccctggcaggaagatggctgtgagggacaggggagtggcgccctgcaatatttgcatgtcgctatgtgttctgggaaatca
ccataaacgtgaaatgtctttggatttgggaatcttataagttctgtatgagaccactctttcccGNNNNNNNNNNNNNNNNNNNNG
TTTCAGAGCTAAGCACAAGAGTGCATAGCAAGTTGAAATAAGGCTAGTCCGTTTACAACTTGAAAA
AGTGGCACCCGAGTCGGGTGCtttttttctgcagtatttagcatgccccacccatctgcaaggcattctggatagtgtcaaaacagc
cggaaatcaagtccgtttatctcaaactttagcattttgggaataaatgatatttgctatgctggttaaattagattttagttaaatttcctgctgaagctc
tagtacgataagcaacttgacctaagtgtaaagttgagacttccttcaggtttatatagcttgtgcgccgcttgggtacctcGNNNNNNNNN
NNNNNNNNNNNGTTTCAGAGCTACAGCAGAAATGCTGTAGCAAGTTGAAATAAGGCTAGTCCGTT
ATCAACTTGAAAAAGTGGCACCGAGTCGGTGCtttttttGCTTTGTTACTTTATAGAAGAAATTTTGAGT
TTTTGTTTTTTTTTAATAAATAAATAAACATAAATAAATTGTTTGTTGAATTTATTATTAGTATGTAAGTG
TAAATATAATAAAACTTAATATCTATTCAAATTAATAAATAAACCTCGATATACAGACCGATAAAACACA
TGCGTCAATTTTACGCATGATTATCTTTAACGTACGTCACAATATGATTATCTTTCTAGGGTTAAGTAA
CGCGTAGGGCCCGGTACTAACCAAACTGGATCTCTGCTGTCCCTGTAATAAACCCGAAAATTTTGA
ATTTTTGTAATTTGTTTTTGTAATTCTTTAGTTTGTATGTCTGTTGCTATTATGTCTACTATTCTTTCCC
CTGCACTGTACCCCCCAATCCCCCCTTTTCTTTTAAAATTGTGGATGAATACTGCCATTTGTCTGCA
GAATTGGCGCACGCAGTGCCGATCCGTTCACTAATCGAATGGATCTGTCTCTGTCTCTCTCTCCA
CCTTCTTCTTCTATTCCTTCGGGCCTGTCGGGTCCCCTCGGGGTTGGGAGGTGGGTCTGAAACG
ATAATGGTGAATATCCCTGCCTAACTCTATTCACTATAGAAAGTACAGCAAAAACTATTCTTAAACCT
ACCAAGCCTCCTACTATCATTATGAATAATTTTATATACCACAGCCAATTTGTTATGTTAAACCAATTC
CACAAACTTGCCCATTTATCTAATTCCAATAATTCTTGTTCATTCTTTTCTTGCTGGTTTTGCGATTC
TTCAATTAAGGAGTGTATTAAGCTTGTGTAATTGTTAATTTCTCTGTCCCACTCCATCCAGGTCGTG
TGATTCCAAATCTGTTCCAGAGATTTATTACTCCAACTAGCATTCCAAGGCACAGCAGTGGTGCAA
ATGAGTTTTCCAGAGCAACCCCAAATCCCCAGGAGCTGTTGATCCTTTAGGTATCTTTCCACAGCC
AGGATTCTTGCCTGGAGCTGCTTGATGCCCCAGACTGTGAGTTGCAACAGATGCTGTTGCGCCT
CAATAGCCCTCAGCAAATTGTTCTGCTGCTGCACTATACCAGACAATAATTGTCTGGCCTGTACCG
TCAGCGTCATTGACGCTGCGCCCATAGTGCTTCCTGCTGCTCCCAAGAACCCAAGGAACAAAGC
TCCTATTCCCACTGCTCTTTTTTCTCTCTGCACCACTCTTCTCTTTGCCTTGGTGGGTGCTACTCC
TAATGGTTCAATTTTTACTACTTTATATTTATATAATTCACTTCTCCAATTG

Annotation of qgRNA features (feature and position in the sequence):

HIV-1 Psi	383..508
5' LTR (truncated)	555..735
CMV enhancer	953..1256
PB3'	3167..3479
bGH poly(A) signal	3492..3716
3'LTR	3742..3919
cppt	3998..4017
TagBFP	4077..4778
T2A	4779..4832
PuroR	4851..5447
PGK promoter	5487..5985
hU6	6018..6267
sgRNA1	6268..6287
tracrRNA 1 (cr1)	6288..6373
EM7 promoter	6381..6428
TpR	6447..6683
mU6	6691..7006
sgRNA2	7007..7026
tracrRNA2 (cr2)	7027..7114
hH1	7122..7345
sgRNA3	7346..7365
tracrRNA 3 (cr3)	7366..7453
h7SK	7461..7704
sgRNA4	7705..7724
tracrRNA (cr4)	7725..7810
PB 5'	7820..8054
cPPT/CTS	8115..8232
RRE	8759..8992

Four sgRNA primer sequence (5'-3', N_{20} in sgRNA1 primer Fwd, sgRNA2 primer Fwd, sgRNA3 primer Fwd is exactly the sgRNA sequence, however, in sgRNA4 primer Rev it should be the reverse complement sequence of the sgRNA sequence):

sgRNA1 primer Fwd: ttgtggaaaggacgaaacaccGN_{20}GTTTAAGAGCTAAGCTG

sgRNA2 primer Fwd: cttggagaaaagccttgtttGN_{20}GTTTGAGAGCTAAGCAGA

sgRNA3 primer Fwd: gtatgagaccactctttcccGN_{20}GTTTCAGAGCTAAGCACA

sgRNA4 primer Rev: ATTTCTGCTGTAGCTCTGAAACN_{20}Cgaggtacccaagcggc

Common primer sequences (5'-3'):

mU6 Rev: CAAACAAGGCTTTTCTCCAAGGG

M Rev: Cgggaaagagtggtctcataca

Constant template sequences (5'-3')

C1 sequence

GTTTAAGAGCTAAGCTGGAAACAGCATAGCAAGTTTAAATAAGGCTAGTCCGTTATCAACTTGAAAA
AGTGGCACCGAGTCGGTGCtttttttgttgacaattaatcatcggcatagtatatcggcatagtataatacgacaaggtgaggaact
aaaccatgggtcaaagtagcgatgaagccaacgctcccgttgcagggcagtttgcgcttcccctgagtgccacctttggcttaggggatcgcgt
acgcaagaaatctggtgccgcttggcagggtcaagtcgtcggttggtattgcacaaaactcactcctgaaggctatgcggtcgagtccgaatcc
cacccaggctcagtgcaaatttatcctgtggctgcacttgaacgtgtggcctaatttttttgatccgacgccgccatctctaggcccgcgccggcc
ccctcgcacagacttgtgggagaagctcggctactcccctgccccggttaatttgcatataatatttcctagtaactatagaggcttaatgtgcgat
aaaagacagataatctgttctttttaatactagctacattttacatgataggcttggatttctataagagatacaaatactaaattattatttaaaaaa
cagcacaaaaggaaactcaccctaactgtaaagtaattgtgtgttttgagactataaatatcccttggagaaaagccttgtttG

M sequence

GTTTGAGAGCTAAGCAGAAAGCTGCATAGCAAGTTCAAATAAGGCTAGTCCGTACACAACTTGAAA
AAGTGGCAGCCGAGTCGGCTGCtttttttgaacgctgacgtcatcaacccgctccaaggaatcgcgggcccagtgtcactaggc
gggaacacccagcgcgcgtgcgccctggcaggaagatggctgtgagggacaggggagtggcgccctgcaatatttgcatgtcgctatgtgtt
ctgggaaatcaccataaacgtgaaatgtctttggatttgggaatcttataagttctgtatgagaccactctttcccG

C2s sequence

GTTTCAGAGCTAAGCACAAGAGTGCATAGCAAGTTGAAATAAGGCTAGTCCGTTTACAACTTGAAA
AAGTGGCACCCGAGTCGGGTGCtttttttctgcagtatttagcatgccccacccatctgcaaggcattctggatagtgtcaaaaca
gccggaaatcaagtccgtttatctcaaactttagcattttgggaataaatgatatttgctatgctggttaaattagattttagttaaatttcctgctgaag
ctctagtacgataagcaacttgacctaagtgtaaagttgagacttccttcaggtttatatagcttgtgcgccgcttgggtacctcG
