# Supplementary material for: Arrayed CRISPR libraries for the genome-wide activation, deletion and silencing of human protein-coding genes
Source: Nat Biomed Eng. 2024 Dec 4;9(1):127–48. doi: 10.1038/s41551-024-01278-4 (PMC11754104; doi:10.1038/s41551-024-01278-4)
Supplement: Supplementary file 2 — Reporting Summary [file 41551_2024_1278_MOESM2_ESM.pdf]

nature portfolio

Corresponding author(s): Jiang-An Yin, Adriano Aguzzi

Last updated by author(s): Aug 11, 2024

Reporting Summary

Nature Portfolio wishes to improve the reproducibility of the work that we publish. This form provides structure for consistency and transparency in reporting. For further information on Nature Portfolio policies, see our Editorial Policies and the Editorial Policy Checklist.

Statistics

For all statistical analyses, confirm that the following items are present in the figure legend, table legend, main text, or Methods section.

n/a	Confirmed	
☐	☒	The exact sample size (*n*) for each experimental group/condition, given as a discrete number and unit of measurement
☐	☒	A statement on whether measurements were taken from distinct samples or whether the same sample was measured repeatedly
☐	☒	The statistical test(s) used AND whether they are one- or two-sided<br>*Only common tests should be described solely by name; describe more complex techniques in the Methods section.*
☒	☐	A description of all covariates tested
☐	☒	A description of any assumptions or corrections, such as tests of normality and adjustment for multiple comparisons
☐	☒	A full description of the statistical parameters including central tendency (e.g. means) or other basic estimates (e.g. regression coefficient) AND variation (e.g. standard deviation) or associated estimates of uncertainty (e.g. confidence intervals)
☐	☒	For null hypothesis testing, the test statistic (e.g. *F*, *t*, *r*) with confidence intervals, effect sizes, degrees of freedom and *P* value noted<br>*Give P values as exact values whenever suitable.*
☒	☐	For Bayesian analysis, information on the choice of priors and Markov chain Monte Carlo settings
☒	☐	For hierarchical and complex designs, identification of the appropriate level for tests and full reporting of outcomes
☐	☒	Estimates of effect sizes (e.g. Cohen's *d*, Pearson's *r*), indicating how they were calculated

Our web collection on statistics for biologists contains articles on many of the points above.

Software and code

Policy information about availability of computer code

Data collection	For cell analysis and sorting with flow cytometers, data were obtained using the BD FACSDiva software. For the construction of our arrayed CRISPR libraries, we wrote a custom code pipeline that is available at https://github.com/Lukas-1/CRISPR_4sgRNA. All code is based on the R statistical programming environment, version 3.6.3, and Bioconductor suite, version 3.10.0. For TR-FRET measurement to determine PrPC level in cell lysates, EnVision Manager software (version 1.14.3049.528) was used. GFP-SQSTM1 images were taken using Zeiss fluorescent microscopy with ZEN 2012 software (version 1.1.2.0); YFP-LC3 images were taken with Leica confocal microscopy with LAS X software (version 4.5.0.25531).
Data analysis	For flow cytometry, data were analyzed with FlowJo (version 10.9.0). For the quality-control analysis with single-molecule sequencing of plasmids and for the arrayed and pooled CRISPRoff screens, the analysis was performed with custom code available at https://github.com/Lukas-1/CRISPR_4sgRNA/tree/master/6)%20Individual%20experiments using the R statistical programming environment, version 3.6.3. Western blot images, GFP-SQSTM1 and YFP-LC3 images were analyzed with Fiji (ImageJ, version 2.14.0). For plotting data and statistical analyses, GraphPad Prism (version 9.4.0) or Microsoft Excel was used.

For manuscripts utilizing custom algorithms or software that are central to the research but not yet described in published literature, software must be made available to editors and reviewers. We strongly encourage code deposition in a community repository (e.g. GitHub). See the Nature Portfolio guidelines for submitting code & software for further information.

Data

Policy information about availability of data

All manuscripts must include a data availability statement. This statement should provide the following information, where applicable:

- Accession codes, unique identifiers, or web links for publicly available datasets
- A description of any restrictions on data availability
- For clinical datasets or third party data, please ensure that the statement adheres to our policy

The complete sgRNA sequences, metadata and annotation for our T.spiezzo and T.gonfio libraries are included as Source Data File 1. The count and fold-change data from the CRISPR screens are available as Source Data Files 2–4. All additional experimental and sequencing data are available from the corresponding authors on reasonable request. Source data are provided with this paper.

Research involving human participants, their data, or biological material

Policy information about studies with human participants or human data. See also policy information about sex, gender (identity/presentation), and sexual orientation and race, ethnicity and racism.

Reporting on sex and gender	The study did not involve human research participants.
Reporting on race, ethnicity, or other socially relevant groupings	-
Population characteristics	-
Recruitment	-
Ethics oversight	-

Note that full information on the approval of the study protocol must also be provided in the manuscript.

Field-specific reporting

Please select the one below that is the best fit for your research. If you are not sure, read the appropriate sections before making your selection.

☒ Life sciences ☐ Behavioural & social sciences ☐ Ecological, evolutionary & environmental sciences

For a reference copy of the document with all sections, see nature.com/documents/nr-reporting-summary-flat.pdf

Life sciences study design

All studies must disclose on these points even when the disclosure is negative.

Sample size	No statistical method was used to predetermine sample sizes. The sample sizes in our experiments were determined according to established literature.
Data exclusions	For the calculation of the mean Z' factors in Extended Data Fig. 2e, one data point of sg1 of CD4 and one data point of sg1 of CD200 were excluded because the values were outliers of the assessment. The criteria was not pre-established. For all other figures or data, no data were excluded.
Replication	All experiments were performed multiple times, usually with 3–5 biological repeats, to reach a conclusive result. Genomic screens were performed 1–2 times and the top candidate genes were validated by various methods. Details on the number of experimental repeats and statistical analysis can be found in the figure legends.
Randomization	All samples were randomized to ensure no bias was introduced by investigators.
Blinding	The investigators were not blinded to the experimental group allocation. Most of the experiments were measured with quantitative readouts such as real-time quantitative PCR, flow cytometry and sequencing. Data analysis was performed while remaining blind to the sample assignments.

Reporting for specific materials, systems and methods

We require information from authors about some types of materials, experimental systems and methods used in many studies. Here, indicate whether each material, system or method listed is relevant to your study. If you are not sure if a list item applies to your research, read the appropriate section before selecting a response.

Materials & experimental systems

n/a	Involved in the study	
☐	☒	Antibodies
☐	☒	Eukaryotic cell lines
☒	☐	Palaeontology and archaeology
☒	☐	Animals and other organisms
☒	☐	Clinical data
☒	☐	Dual use research of concern
☒	☐	Plants

Methods

n/a	Involved in the study	
☒	☐	ChIP-seq
☐	☒	Flow cytometry
☒	☐	MRI-based neuroimaging

Antibodies

Antibodies used

Flow cytometry: APC anti-human CD47 Antibody (Biolegend, Cat. # 323124, Clone CC2C6); PE anti-human CD119 (IFN-γ R α chain) Antibody (Biolegend, Cat. # 308606, Clone GIR-208); APC anti-human CD146 Antibody (Biolegend, Cat. # 361016, Clone P1H12); PE anti-human CD29 Antibody (Biolegend, Cat. # 303004, Clone TS2/16); FITC anti-human CD81 (TAPA-1) Antibody (Biolegend, Cat. # 349504, Clone 5A6); APC anti-human CD151 (PETA-3) Antibody (Biolegend, Cat. # 350406, Clone 50-6), APC anti-human CD2 Antibody (Biolegend, Cat. # 300214, Clone RPA-2.10); APC anti-human CD4 Antibody (Biolegend, Cat. # 357408, Clone A161A1); APC anti-human CD200 (OX2) Antibody (Biolegend, Cat. # 329208, Clone OX-104); FITC Anti-EpCAM antibody [VU-1D9] (Abcam Cat. # ab112067); Alexa Fluor® 647 anti-mouse/human CD44 Antibody (Biolegend, Cat. # 103018, Clone IM7).

PrPC screen and validation: The anti-human PrPC antibodies POM1 and POM2 were produced in-house. The EU-POM2 antibody was produced by conjugating the POM2 antibody with europium (Eu, Perkin Elmer, Cat.# AD0013) in-house; the APC-POM1 antibody was produced by conjugating the POM1 antibody with allophycocyanin (APC, Abcam, Cat.# ab201807) in-house; Recombinant Anti-Vinculin antibody [EPR8185] (Abcam, Cat.# ab129002).

Autophagy hits validation: LC3B (D11) XP® Rabbit mAb #3868 (Cell Signaling, Cat. # 3868); Anti-GAPDH antibody produced in rabbit (Sigma-Aldrich, Cat. # G9545).

Validation

All commercial antibodies were validated by their suppliers: Biolegend, Abcam, Cell Siganling, and Sigma-Aldrich. Anti-PrPC antibodies including EU-POM2, APC-POM1, and POM2 were well established and validated in the Aguzzi laboratory and used in previous publications (Heinzer et al., PLoS Pathog 17, e1010013 (2021); (Pease et al., Brain Pathol 29, 232-244 (2019)).

Eukaryotic cell lines

Policy information about cell lines and Sex and Gender in Research

Cell line source(s)

HEK293, H4, HEK293T, GIMEN, THP-1, ARH-77, and HCT116 cells were purchased from ATCC. U251-MG was purchased from Kerafast, Inc., Boston, MA, USA, AccessionID: CVCL_0021. iPSC cell line (Gm23280) was obtained from the Coriell Institute for Medical Research (https://www.coriell.org) and iNeurons were differentiated from the iPSCs. Kidney organoids were differentiated from NPCs which were established at the Novartis Institutes for Biomedical Research.

Authentication

Each cell line was handled and cultured separately, stored at early passages, and discarded after more than 20 passages. All these measures helped to preserve cell identity.

Mycoplasma contamination

iPSCs tested negative for mycoplasma once per month. All other cells were not tested for mycoplasma contamination.

Commonly misidentified lines
(See ICLAC register)

No commonly misidentified cell lines were used.

Plants

Seed stocks

The study did not involve plants.

Novel plant genotypes

-

Authentication

-

Flow Cytometry

Plots

Confirm that:

☒ The axis labels state the marker and fluorochrome used (e.g. CD4-FITC).

☒ The axis scales are clearly visible. Include numbers along axes only for bottom left plot of group (a 'group' is an analysis of identical markers).

☒ All plots are contour plots with outliers or pseudocolor plots.

☒ A numerical value for number of cells or percentage (with statistics) is provided.

Methodology

Sample preparation	Suspension cells were directly collected via centrifugation. All other cells were dissociated with trypsin and then collected via centrifugation. Cells were washed once with PBS and analyzed in 500 µl of PBS. For antibody staining conditions, live cells were stained with the respective antibodies according to the manual and then analyzed. Non-treated cells were used as controls.
Instrument	Cells were analyzed using BD Canto II or LSR II Fortessa flow cytometers. Cells were sorted by BD FACSymphony S6 or FACSAria III.
Software	FACS data were collected using BD FACSDiva (BD Biosciences), and data analysis was performed using FlowJo (version 10.9.0). Statistical analysis and data visualization were conducted using GraphPad Prism (version 9.4.0).
Cell population abundance	Cell sorting was applied in the autophagy screen to isolate GFP-high and GFP-low cell populations. Cell sorting was also applied in CRISPRoff screens to enrich cells that were double positives for mScarletI and GFP (using the dual-guide CRISPRoff library) or mScarletI and TagBFP (using the T.gonfio library), respectively. For confirmation, a subsample of the sorted cells was reanalyzed using the same parameters with the corresponding sorters.
Gating strategy	Please refer to the Supplementary Fig. 1.

☒ Tick this box to confirm that a figure exemplifying the gating strategy is provided in the Supplementary Information.
